# Interpretation of mRNA splicing mutations in genetic disease: review of the literature and guidelines for information-theoretical analysis

**DOI:** 10.12688/f1000research.5654.1

**Published:** 2014-11-18

**Authors:** Natasha Caminsky, Eliseos J. Mucaki, Peter K. Rogan

**Affiliations:** 1 Department of Biochemistry, Schulich School of Medicine and Dentistry, Western University, London, ON, N6A 2C1, Canada; 2 Departments of Biochemistry and Computer Science, Western University, London, ON, N6A 2C1, Canada

**Keywords:** mRNA, splicing, mutation, genetic disease, rare disease

## Abstract

The interpretation of genomic variants has become one of the paramount challenges in the post-genome sequencing era. In this review we summarize nearly 20 years of research on the applications of information theory (IT) to interpret coding and non-coding mutations that alter mRNA splicing in rare and common diseases. We compile and summarize the spectrum of published variants analyzed by IT, to provide a broad perspective of the distribution of deleterious natural and cryptic splice site variants detected, as well as those affecting splicing regulatory sequences. Results for natural splice site mutations can be interrogated dynamically with Splicing Mutation Calculator, a companion software program that computes changes in information content for any splice site substitution, linked to corresponding publications containing these mutations. The accuracy of IT-based analysis was assessed in the context of experimentally validated mutations. Because splice site information quantifies binding affinity, IT-based analyses can discern the differences between variants that account for the observed reduced (leaky) versus abolished mRNA splicing. We extend this principle by comparing predicted mutations in natural, cryptic, and regulatory splice sites with observed deleterious phenotypic and benign effects. Our analysis of 1727 variants revealed a number of general principles useful for ensuring portability of these analyses and accurate input and interpretation of mutations. We offer guidelines for optimal use of IT software for interpretation of mRNA splicing mutations.

## Introduction

Pre-mRNA splicing is a necessary step in the production of a functional protein product. It consists of the recognition of intron/exon boundaries, and the subsequent excision of the introns. It is important to distinguish between alternate splicing isoforms and mutant splice forms. The former consists of using different combinations of splice sites for the same gene. It is estimated to occur in over 60% of human genes, some of which will have multiple alternate isoforms ^1, 2^. For example, *NF1* is reported to produce 46 splice variants ^3^. The cell regulates this naturally occurring process through the availability of tissue-specific splice factors. Alternative splicing is not generated by changes in the unspliced RNA sequence, whereas mutations that produce non-constitutive splice forms are the result of dysregulation of natural splice site recognition. Mutations can have various consequences to RNA processing, such as exon skipping, cryptic splicing, intron inclusion, leaky splicing, or less frequently, introduction of pseudo-exons into the processed mRNA. A broad range of molecular phenotypes are possible depending on the type and severity of the mutation, making it imperative to understand the consequences of splicing mutations. For the purposes of this review, we consider sequence changes in genes that affect transcript structure or abundance to be mutations, regardless of their allele frequencies. Although spliceosomal recognition and RNA binding factors are operative in mutation-derived and normal alternative mRNA splicing events, this review is focused on aberrant sequence changes that alter constitutive splicing, and often result in clinically abnormal phenotypes.

The process of U1/U2-based mRNA splicing involves the recognition of a number of key sequence components ^4, 5^, with exons defined by both intronic and exonic features ^4, 6^. The exonic and intronic sequences flanking the 5´ end of an intron is termed the donor site and the 3´ end, the acceptor site. In typical mRNA splicing, the natural donor and acceptor splice sites span intervals of 10 and 28 bases in length, respectively. It is a common misconception that these sequences (especially the dinucleotides immediately intronic to the exon) are invariant. Although highly conserved, these sequences vary at different splice junctions within a gene as well as between genes. The particular combination of nucleotides at each position within the same splice site determines its overall strength, which dictates the likelihood of recognition by the U1 and U2 spliceosomes.

In addition, binding sites for splicing regulatory elements have been shown to reside over a range of distances from the corresponding natural splice sites ^7^; the impact of these sites appears to be related to their binding affinities to the cognate RNA binding proteins and to their distance from the proximate intron/exon boundary ^8^. Recognition sites for these regulatory proteins can reside either within introns or exons. Those within exons are commonly referred to as exonic splice enhancers or silencers (ESE or ESS, respectively), whereas the corresponding designations for intronic elements are ISE or ISS. Sequence variants affecting these protein-binding sites (or mutations in the binding proteins themselves) have been documented as contributing to aberrant splicing and pathogenic phenotypes. We focus on variants occurring in *cis* with target genes, as opposed to those in the splicing complex (in *trans)*, leading to abnormal splicing. The efficiency and specificity of splicing depends on the combination of natural splice site strengths and the binding of splicing regulatory proteins that orchestrate exon recognition ^9^.

Mutations that affect pre-mRNA splicing account for at least 15% of disease-causing mutations ^10^ with up to 50% of all mutations described in some genes ^11, 12^. Interpreting the effects that these variants have on splicing is not straightforward because natural and regulatory splice sites exhibit considerable sequence variation. Furthermore, performing *in vitro* experiments to verify the consequences of each variant is costly and time consuming, and may not be practical. *In silico* prediction methods have become essential resources for analyzing these variants. Software programs for splicing analysis use a wide variety of bioinformatic approaches. Several splice site prediction tools compare the predicted mutant sequence to a consensus sequence, based on a set of functional acceptor or donor splice sites ^13^. A drawback of this approach is that low-frequency nucleotides present in functional splice sites are not represented, which can lead to misinterpretation and false-positive mutation predictions. One example of this was illustrated by Rogan and Schneider (1995), in which the variant, IVS12-6T>C in *MSH2*, described by Fishel *et al*. (1993) was predicted to be benign, despite being located 6 nt from the natural acceptor splice junction ^14, 15^. The consensus sequence fails to indicate that C and T at this position are nearly equally probable, which reclassified this transition as a polymorphism rather than a pathogenic variant. This conclusion is supported by evidence that ~10% of normal individuals without predisposition to non-polyposis colon cancer harbour this alternate allele ^16^.

Over the last 20 years, we and others have developed an information theory (IT)-based approach for prediction of splicing mutations, and their impact on mRNA structure and abundance. The effects of these mutations is founded on the formal relationship between IT and the second law of thermodynamics, in that the change in information ascribed to a sequence variant within a splice site is directly related to thermodynamic entropy and free energy of binding ^17, 18^. A weight matrix consisting of the Shannon information (product of the probability of each nucleotide and –log _2_ of its probability) at each position of the splice site is constructed. The individual information for a splice site ( *R _i_*, in bits) is defined as the dot product of this weight matrix and the unitary vector of a particular splice site sequence. The magnitude of the information content of a nucleotide within a given site is an indication of its level of conservation relative to a set of functional sites. This method retains all of the sequence variability inherent in each model of donor and acceptor splice sites. By contrast, each base in the consensus sequence has the maximum *R _i_* value, which is actually rare in the human genome, and is generally not representative of the preponderance of natural splice sites. Prior to the introduction of IT-based approaches, consensus sequence-based methods were widely used ^13^. Also, the use of neural networks, trained on sequences experimentally determined to be “bound” and “unbound”, was another early approach used to predict splice sites ^19^. However, these unbound set of sequences are known to harbour some contaminating functional sites ^20, 21^, which can limit the sensitivity and specificity of these networks ^22^.

There are instances when IT does not accurately predict the consequences of a splice variant. This can often be attributed to instances involving multiple sites or multiple regulatory factors, which are not components of current splicing models. In addition, splicing regulatory proteins can share overlapping and degenerate binding sites, and may exert conflicting effects (for example, serine-arginine [SR] vs. hnRNP proteins), making *in silico* prediction less reliable and accurate in these cases ^23^. Finally, functional cryptic splicing motifs occurring deep within the introns can be challenging to identify, because they tend to be less well conserved than natural splice sites ^24, 25^.

Nevertheless, a number of authors have recommended IT methods for analysis of splice site variants (N = 29; Supplementary Table 1). In fact, this approach has been described as equivalent to using a general reference textbook as a diagnostic tool, which complemented by functional assays, may provide a complete molecular diagnosis ^26^. Most of the applications of IT for splicing mutation analysis have involved predominantly rare diseases, as well as some low frequency variants associated with more common genetic conditions. This is because IT has been used to assess how well computed changes in binding affinity conform to levels of expression and/or patient phenotypes.

Many IT studies have focused on sequence variants in individual disorders or genes. Our synopsis of the broader implications of this work sets the stage for this compilation of peer-reviewed variants with accompanying IT analyses. We cover all publications retrieved through PubMed and Google Scholar that cite the use of IT (N = 367; Supplementary Bibliography) before September 2014. These items include primary research articles, review articles, presentations, and theses. Of all references, 216 publications reported variants or other results or analyses pertinent to this review ( Supplementary Table 2). In the remaining studies, analyses were either not performed, insufficient information was provided to reproduce the reported result, or authors described unrelated applications of IT-based analysis. We summarize the spectrum of variants analyzed to obtain a global perspective of splicing mutations resulting in genetic disease. We also highlight common errors that can occur in variant analysis and interpretation, and offer guidelines for optimal use of our software programs for interpretation of splicing mutations.

## Information theory and splice site analysis

IT was first introduced by Claude Shannon in 1948 and is now used in a variety of disciplines to express the average number of bits (i.e. the information content) needed to communicate symbols in a message ^27^. Bits are the basic unit used in computing and can have one of two values (typically the answer to a yes/no, true/false, or +/- problem). In nucleic acid molecular biology, the symbols in the message comprise a group of related, aligned sequences, with the average number of bits in the set corresponding to the amount of information in the message. This is determined from the information content at each position in the sequence, summed over all positions ^28^. The average information is depicted graphically by a sequence logo, which stacks the individual nucleotides at each position ranked by frequency, and where the height of the stack is the position-specific contribution to the average information ^29^. If the set of sequences are functional binding sites recognized by the same factor, the individual information in each site (i.e. *R _i_* value) is related to thermodynamic entropy, and thus, to the free energy of binding ^18^.

**Figure 1.**
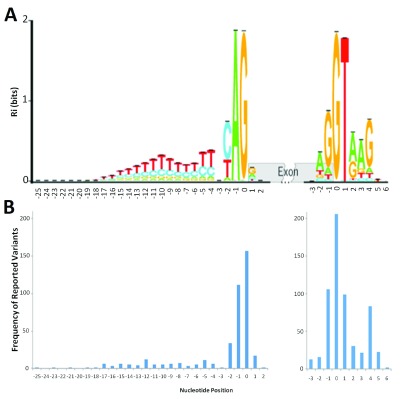
Distribution of deleterious natural site variants relative to information content. **A**) The sequence logo for human acceptor and donor splice sites based on the positive (+) strand of the October 2000 (hg5) genome draft. The logo shows the distribution of information contents ( *R _i_* in bits) at each position over the region of 28 nucleotides for acceptor [-25, +2] and 10 nucleotides for donor [-3, +6] from the first nucleotide of the splice junction (position 0). Nucleotide height represents its frequency at that position. The horizontal bar atop each stack indicates the standard deviation at that position. This figure was modified from Rogan *et al.* (2003) to include splice sites in genes on both strands of the annotated human reference genome ^30^. **B**) The distribution of deleterious single-nucleotide variants reported at the natural acceptor (left) and donor (right) splice sites. The variants used to populate this graph ( Supplementary Table 8) were included only if they were reported to negatively affect splicing (N = 419 for acceptors, 599 for donors). The image was aligned to the sequence logo ( **A**) to illustrate potential correlation of number of splicing variants at a position to the information content at that position.

The information content of a nucleic acid binding site is related to the affinity of its interaction with proteins and other macromolecular complexes, such as the case during mRNA splicing ^18^. Information theory-based position weight matrices (PWM; *R _i_* [b,l] - also referred to as a ribl - where b and l correspond to the nucleotide and position in the splice site) can be determined for set of known binding sites, in this case, for the purpose of calculating individual and average sequence information ^28^. Figure 1 shows an example of sequence logos for the canonical acceptor (or 3´, recognized by the U2 spliceosome) and donor (or 5´, recognized by the U1 spliceosome) splice sites, computed from the majority of constitutive sites at annotated splice junctions in the human genome ^30^. The information contained within the natural splice donor site is distributed between the last codon of each exon and the adjacent 6 nucleotides of intronic sequence, whereas the acceptor sites are almost entirely intronic, extending 26 nucleotides upstream from the exon boundary.

The distributions of *R _i_* values for these sets are approximately Gaussian, with a couple of important exceptions, namely the distribution has defined upper and lower bounds ^18^. The upper limit corresponds to the consensus sequence, as it is not possible to have stronger binding than an exact match to this sequence. The theoretical lower limit corresponds to *R _i_* = 0 bits. An *R _i_* value less than zero implies that energy would be required (Δ *G* > 0 kcal/mol) for a stable binding complex to form, i.e. that the event would not occur spontaneously without an exogenous source of energy. The minimum strength site is zero bits, the equilibrium state (Δ *G* = 0). Assuming the contacts at each position in the same binding site form independently, this approach is accurate and quantitative. Altering a nucleotide with high information (implying high prevalence and conservation at that position) will have a greater impact on binding, than if a less-well conserved base were altered. The change in information due to a mutation in a site (Δ *R _i_*) is the difference between *R _i,final_* and *R _i,initial_* values, where *R _i,final_* is the information of the sequence containing the variant, and *R _i,initial_* the information of the reference (wild-type) sequence. The minimum fold change in binding affinity resulting from the mutation is an exponential function based on Δ *R _i_*, or ≥ 2 ^Δ *Ri*^ (Ref. ^18^).

## Software resources

### Delila package/system

Information analysis was originally performed using the Delila sequence analysis system, which included a language to process nucleic acid sequences, and a library of sequence tools to retrieve and process various types of sequence data ^31, 32^. Tools to measure information content of nucleic acid sequences were subsequently added to Delila ^28^. Initially, models of information content of bacteriophage T7 RNA polymerase binding sites and other bacterial control systems were studied, and mRNA splice sites were subsequently developed ^28, 33^. Later, tools to display binding sites as sequence logos of average information, and sequence walkers showing individual information were incorporated into Delila ^20, 29^. The Automated Splice Site Analysis (ASSA) server introduced in 2004, and its successor, Automated Splice Site and Exon Definition Analysis server ( http://splice.uwo.ca; ASSEDA), have been freely available throughout the last decade, and have been used for IT-based calculations on nucleic acid sequences for the preceding 20 years ^34, 35^. Both ASSA and ASSEDA still use the Delila program suite to retrieve sequences, calculate information content, and create sequence walker representations of individual binding sites.

### ASSA/ASSEDA

To simplify mutation analysis, we built a web interface for variant analysis using Delila software as the processing backbone ^34^. Our aim was to standardize and facilitate IT-based mutation analysis by using Human Genome Variation Society (HGVS)-approved variant nomenclature (which has since become the worldwide standard), employing server-based retrieval/processing, and reporting results as concise predictions in both tabular and sequence walker display formats. Initially, ASSA results described mutations in relation to genome annotations from the first finished genome release (hg15) ^34^. While many publications cited this version of ASSA for novel splicing mutation analysis, continued improvements have introduced more accurate reference sequences, annotations, and models (for both constitutive and regulatory splice sites) based on more comprehensive sets of binding sites. The ASSA server contained the original donor and acceptor information position weight matrices derived by manual curation of GenBank entries ^33^, murine donor and acceptor weight matrices, a subset of splicing enhancer elements (SF2/ASF, SC35 and SRp40), and the lariat branch point recognition sequence ^33^. ASSA reported the strengths of all potential sites predicted within the window selected by the user, highlighted those with the largest changes in *R _i_*, and computed the minimum fold change in binding affinity for each mutation or polymorphism. Tabular results were colour-coded. Unaltered sites above and below the *R _i,min_* (described in Minimum splice site information content and exceptions) were highlighted grey and white, respectively. Pre-existing sites abolished by the variant (where *R _i,final_* < *R _i,min_*) were marked in red, while leaky natural sites ( *R _i,final_* ≥ *R _i,min_*) were highlighted in blue. Cryptic sites that were created, strengthened, or weakened were highlighted in pink, green and teal, respectively. The server parsed any mutation type described precisely by the HGVS notation, including substitutions, insertions, deletions, and combinations of these changes ^36^. Recapitulating variants described in articles before these guidelines were widely adopted proved to be time-consuming and error-prone ^22^. Multiple binding factors had to be analyzed simultaneously; however, results were reported independently. The analysis did not consider other factors relevant to splice site recognition, such as the resulting exon size, or potential formation of cryptically spliced exons.

ASSEDA, the successor software to ASSA, provides a new isoform-oriented type of mutation interpretation, updates the coordinate system to HG19 (GRCh37), adds current gene and single nucleotide polymorphism (SNP) annotations (dbSNP135), and provides additional ribls for other splicing regulatory sites (SRp55, TIA1, ELAVL1, hnRNP A1, hnRNP H, and PTB). All models, except those for SRp55 and hnRNP H, have been built using sequences from publicly available CLIP-seq data, and are based on a larger number of binding site sequences. They have been tested by comparing predictions to validated binding sites from published primary literature, and to any splice-altering variants found within them ^35^. ASSEDA introduces *in silico* exon definition analysis by computing the total splicing information across an exon ^35^. Total exon information ( *R _i,total_*) is the sum of the corresponding donor and acceptor *R _i_* values, and corrected for the gap surprisal term, which is based on the length of the potential exon formed using those sites (from RefSeq) ^37^. The gap surprisal function is based on the genome-wide distribution of constitutive exon lengths, also known as self-information. This term ensures that exons are computationally defined using donor and acceptor splice sites in close proximity ^37, 38^.

Exons of uncommon length lead to large negative gap surprisal terms, which reduces *R _i,total_*. When applied to predicted exons that activate a cryptic splice site, comparison of *R _i,total_* values can more accurately predict cryptic site use than the strength of this site alone. The gap surprisal term decreases the predicted *R _i,total_* value of particularly long internal exons (eg. the 3.4 kb long exon 11 of *BRCA1*; *R _i,total_ =* 1.4 bits), which tends to compensate for this effect with strong splice sites and other sequence elements that enhance natural splice site recognition and suppress internal cryptic splice sites.

The exon definition paradigm extends to the assessment of the impact of mutations in ESE/ISS elements. ASSEDA calculates *R _i,total_* by adding the *R _i_* value of a regulatory splicing element to the contributions of constitutive splice sites, and applying a second gap surprisal term based on the frequency of distance from the splicing element to the nearest natural site. Currently, the effect of only a single splicing factor can be evaluated by the software, although the approach itself is generalizable to multiple regulatory binding sites. If a variant causes changes in the *R _i_* values of multiple sites, such as the simultaneous creation of both splicing enhancer and repressor elements, there will be less confidence in ASSEDA’s predictions.

Two distinct sets of IT-based models for donors and acceptors are available on ASSEDA. The manually curated ribls were originally determined from 1799 donor and 1744 acceptor sites ^33^. We subsequently derived a set of ribl matrices from genome-wide exon annotations ^30^. These models were automatically curated using the criteria that enforced *R _i_* > 0 for correctly annotated sites. The resultant models consisted of 108,079 acceptor and 111,772 donor splice sites, however these were not formally implemented on the ASSA server until 2011 ^30^. These genome-wide models are used in the calculation of *R _i,total_* values. The Δ *R _i_* values for a single nucleotide splicing variant are similar for both sets of models. Variants having opposite predicted effects between the respective donor or acceptor ribls have not been reported. In general, the genome-wide models report slightly lower information contents, however the frequencies of nucleotides at the 5´ end of the acceptor site differ significantly. This results in differences in the weights in the -4 to -20 nt region between the manually-curated and the genome-wide acceptor ribl matrix, which can significantly lower *R _i_* values based on the genome-wide model. In the genome, thymine is more prevalent than cytosine at these positions and has a higher positive contribution to the overall *R _i_.* This can account for up to a 1.97 bit difference between the models. Guanine nucleotides within this sequence window significantly lower the *R _i_* values computed from the genome-wide acceptor ribl, as well. While these differences contribute only a 0.1–0.4 bit difference to the *R _i_* per nucleotide, the cumulative effect of multiple differences within this window can lead to significant differences between the acceptor *R _i_* values.

### Shannon Pipeline and Veridical

High-throughput DNA sequencing is generating a deluge of novel variants in patients with genetic diseases, most of which currently have unknown significance (VUS). For example, 20% of the patients with Pelizaeus-Merzbacher disease possess VUS, among which are single or compound heterozygous, rare pathogenic mutations ^39^. Many solutions have been proposed, however prediction of pathogenicity by bioinformatic analyses is often inaccurate ^40^. The Shannon Human Splicing Mutation Pipeline software predicts mutations at genome scale to predict which variants may alter mRNA splicing and is based on the same principles and IT models used in ASSA and ASSEDA ^41^. However, this software processes ~5 million substitutions and/or indels in 10–15 minutes. While initially only available for the CLC-Bio Genomics platform, this software is now offered as a web service ( http://shannonpipeline.cytognomix.com). Variants are batched in standard variant call format (VCF). The pipeline reports any genic variant that affects a known natural site or a cryptic site where *R _i,initial_* or *R _i,final_* are ≥ 0 bits and Δ *R _i_* ≥ 1.0 bits, however more stringent criteria for selecting variants with significant information changes can be applied.

In Shirley *et al*. (2013), all variants from the complete genomes of three cancer cell lines (A431, U2OS, U251; N = 816,275) were analyzed ^41^. Variants that were common (≥ 1%) were removed. Variants that weakened natural sites, or strengthened cryptic sites to levels comparable to or exceeding the strength to the nearest natural site, were flagged. Variants that strengthen a natural site could have an effect on the splicing profile of a gene (i.e. reduce the frequency of exon skipping for the corresponding exon), but are less likely to cause a deleterious phenotype. The overall fraction of mutations flagged, after filtering out distant cryptic sites and small Δ *R _i_* values, averaged 0.016%, illustrating how the software can be used for prioritizing variants. Some of the prioritized variants occurred in genes with known defective functional and biochemical pathways in these cancer cell types, i.e. cytokine signalling (in A431), DNA replication and cell cycle (in U2OS). Natural splice mutations were confirmed by expression data to a greater extent than either leaky or cryptic splice site variants.

In a complete cancer cell line genome, the number of cryptic sites with altered *R _i_* values greatly exceeds the number of affected natural splice sites. Many of these are weak decoys, which can occur throughout genes. Using the principle that novel cryptic sites that are likely to be activated must compete with the natural splice site for spliceosomal recognition, the relevant cryptic sites are restricted to those with *R _i_* values comparable to or greater than the corresponding strength of the adjacent natural site of the same polarity ^22^. Additionally, the proximity of potential cryptic sites to the natural site should be considered in assessing whether an exon could be formed with the natural splice site of opposite polarity. Cryptic sites that are considerably weaker than the nearest natural site of the same type, or cryptic sites that would lead to unusually large exons, diminish the likelihood of cryptic site activation. Benaglio *et al.* (2014) used the Shannon Pipeline to screen 303 sequenced patients and flagged five variants that each strengthened or created a different cryptic site ^42^. While comparable in strength to the natural site, these were all distant (>400 nucleotides away) and thus, less likely to be recognized. The authors also stated that the Δ *R _i_* values for three of these sites were discordant with results obtained with NNSplice, a neural network-based splicing prediction program. In fact, both the Shannon Pipeline and NNSplice demonstrated strengthening of these decoy cryptic splice sites.

Shirley *et al*. (2013) evaluated the predictions of the Shannon Pipeline by manually inspecting RNAseq data for each variant with significant information changes in each cell line ^41^. However, manual review is unfeasible for many large datasets, especially from tumors, because of the large numbers of potential somatic mutations affecting splicing in each genome. Veridical, an *in silico* method for validation of DNA sequencing variants that alter mRNA splicing, has been developed to provide high throughput, statistically-robust unbiased evaluation based on RNAseq data ^43^. The method has been implemented as software for analysis of potential splicing variants from large datasets and catalogues their effects. Veridical takes Shannon Pipeline output from predicted genomic variants with effects on splicing and performs a case-control analysis of corresponding expressed transcripts that cover the same genomic region, taken from normal tissues. Upon Yeo-Johnson transformation of the expressed read count distribution, parametric statistics are used to compare normal and abnormal mRNA species (exon skipping, intron inclusion, and cryptic site use). Veridical is designed to be used with large data sets, as the statistical analysis gains power with increasing numbers of control samples. A recent study of 442 breast cancer tumors from the Cancer Genome Atlas Project revealed 5,206 putative splicing mutations using the Shannon Pipeline. Veridical was then used to confirm exon skipping, leaky or cryptic splicing of 988 of these variants ^44^.

## Natural sites

The early splice site recognition literature often oversimplified the composition of the U1/U2-type 5´ donor and 3´ acceptor sites by presenting only consensus sequences and truncating the positions in each site ^13, 45, 46^. However, the conserved tracts extend well beyond the canonical GT and AG dinucleotides adjacent to intron/exon junctions. Furthermore, a small, albeit significant, proportion of natural donor sites (~800, or 0.7%) contain cytosine at position +2 in the genome. This is reflected by a corresponding small decrease in average information at this position ( Figure 1). Sequences adjacent to these positions are more variable, but are nevertheless essential for the accurate recognition by the spliceosome. Specifically, the donor site is defined by the three terminal nucleotides of each exon and the first seven bases of the downstream intron. Conversely, acceptor sites are represented by the first two bases of the exon and the last 26 bases of the upstream intron. Because ASSA and ASSEDA use an integer-based coordinate system, there is a zero coordinate at the first intronic base of each splice site ( Figure 1), which is not used in the conventional numbering system. The coordinate ranges for the donor and acceptor site positions are therefore [-3, +6] and [-25, +2], respectively. Individual information analysis computes the *R _i_* values over these intervals for normal and variant-containing splice sites. As discussed below, information content present in intronic intervals justifies sequencing and analyses of sequences well beyond the locations of the splice junctions themselves.

Certain variants within donor and acceptor sites are tolerated and may even have benign effects, while others have a deleterious impact on spliceosomal recognition. IT accounts for all of these possible outcomes. Unusual donor sites (i.e. with cytosine at position +2) are detected by information analysis, but could be falsely called deleterious by consensus sequence-based methods. Although the terminal position of exons contributes significantly to donor splice sites with a preference for G, a significant proportion of sites naturally possess A or U at this position, or less frequently, C.

Of the published IT-based variant analyses, single nucleotide variants (SNVs) that were reported to affect a natural splice site (multi-nucleotide and insertion/deletion variants are listed separately in Supplementary Table 3) were compiled and reanalyzed. After reducing this set to only those variants occurring within the intervals covered by the splice site information weight matrices described above, 1152 SNVs were reported to affect the strengths of either natural donor or acceptor sites. A variant was considered deleterious if it was predicted to affect splicing (either leaky expression or exon skipping), or if it was experimentally shown to reduce or abolish splicing of the corresponding exon. In instances where prediction and validation did not concur, the latter were used to determine the effect of the variant. Variants predicted to have a neutral effect but demonstrated to be deleterious in the validation study were classified as damaging. In total, 1010 deleterious natural splice site variants were analyzed ( Supplementary Table 4).

Sequence conservation has long been considered a surrogate measure of evolutionary constraint and, by inference, functional significance. The average information quantitates the relative conservation at each of the positions within a binding site. We compiled the mutation spectra for all mutations that significantly affected the strengths of donor and acceptor splice sites and compared these with the average information contents at each position. The panels in figure 1b respectively indicate, at each position of the natural acceptor and donor sites, the frequencies of variants deemed deleterious by information analysis. Interestingly, when the sequence logo is overlaid with the histogram of the corresponding mutation spectra, the relative frequencies of deleterious mutations and the average information are comparable. Indeed, these frequencies and the information contents across each type of site are strongly correlated (r=0.95 for acceptors and 0.89 for donors). Our interpretation is that the susceptibility to deleterious mutation at a position is related to its overall conservation within the splice site, which reflects the contribution of that ribonucleotide to the stability of the interaction with the corresponding spliceosome. Nevertheless, there is an unstated bias in ascertainment in these mutation spectra. Variants occurring at sites with low information and/or that are benign are underrepresented, as they are less likely to be associated with genetic disease, and were less likely to be reported. Also, the distribution is dependent on the region sequenced by the authors of the reviewed publications; in early work, the full sequence interval containing the entire splice site was sometimes not included or unavailable for analysis.

**Figure 2.**
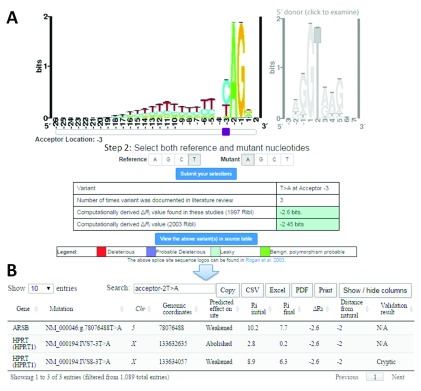
Sample retrieval of average change in information content (Δ *R _i_*) with splicing mutation calculator (SMC) for published mutations. **A**) Example mutation input for SMC (T>A at the 3 ^rd^ intronic position of natural acceptor). The type of splice site is selected by clicking on the corresponding sequence logo (acceptor [left] or donor [right]). The purple slider bar appearing below the logo is used to select the position of the mutation. The reference and mutant nucleotides are then designated, and the variant is submitted to the software (‘Submit your selection’). SMC outputs a table indicating the user input, the number of instances in the literature where this substitution has been analyzed using IT, and the computed Δ *R _i_* values (in bits) using both the old (1992; top) and new (2003; bottom) ribls. The cell color for Δ *R _i_* values indicates the predicted severity of the inputted variant according to defined thresholds ^22, 168^. **B**) Tabular output detailing each instance of the selected mutation from the source table. The user may view, in a separate window, extensive details of all variants referred to in SMC output ( Supplementary Table 10).

An interactive website was created to summarize this set of SNVs. This software application renders interpretations of variant effects in a more practical, useful way than the corresponding table of supplemental data ( Supplemental Table 10). The “Splicing Mutation Calculator” (SMC; http://splicemc.cytognomix.com) is a web service that amalgamates all published results for the same type of substitution in a natural splice site, regardless of genic context. Variants that create cryptic splice sites were not included, because we consider these cases to be sequence-specific as opposed to positional. With this program, users have the option of exploring mutation data (at present, only SNVs can be analyzed) linked to the original literature citations. SMC processes and provides literature support for the variants that occur within the defined regions spanned by natural splice sites. The user first selects the type of site (donor or acceptor), position (based on ASSEDA’s integer-based system), wild-type or reference nucleotide, and the alternate substitution at that position ( Figure 2a). The software tool outputs the Δ *R _i_* and the number of variants that have been reported and analyzed to date using IT ( Figure 2b). SMC provisionally classifies the reported variants based on the degree to which these predicted effects are expected to decrease spliceosomal affinity, and consequently splicing. The following criteria are empirically based on affinity changes and a summary of published phenotypes associated with these changes: “Deleterious” (if the site is weakened by more than 7.0 bits), “Probably Deleterious” (if the site is weakened such that -4.0 bits ≥ Δ *R _i_* ≥ -7.0 bits), “Leaky” (the site is weakened such that -1.0 bits ≥ Δ *R _i_ ≥ -*4.0 bits), or “Benign, probable polymorphism” (if the site is weakened by less than 1.0 bits). In this first release of SMC, we have omitted “benign” variants, which are likely polymorphisms; these will be catalogued and included in a later version. It is important to appreciate that the Δ *R _i_* is a constant for a specific nucleotide change at a specific position, though the absolute strength of the splice site depends on the sequence context of the mutation. This context varies between mutations, and *R _i,initial_* is not the same for each case, which can result in different *R _i,final_* values for different mutations.

Besides published sources, the software also can predict effects of mutations by computing Δ *R _i_* values directly. Particular substitutions that have not been reported in Supplemental Table 10 can nonetheless be provisionally interpreted. The Δ *R _i_* value is computed and reported from the ribl. While SMC enables rapid exploration of results for validated and novel mutations, it is, however, not a replacement for ASSEDA or the Shannon Pipeline, since it does not consider the sequence context, which can also influence the interpretation of deleterious, leaky, or benign variants.

### Minimum splice site information content and exceptions

The minimum theoretical information content of a binding site, *R _i,min_*, is zero bits ^18^. Comparison of the *R _i,_* values of a series of inactivated and minimally active splice sites revealed the minimum strength of functional splice sites ( *R _i,min_*) to be at least 2.4 bits for the original donor and acceptor models of Stephens and Schneider (1992) (based on 103 mutations with functional validation, including 57 natural and 46 cryptic site activating mutations) ^22^. This value was redefined based on information models from a genome-wide set of donor and acceptor models ( Figure 1a) to be 1.6 bits using the identical set of mutations ^30^. It is likely that the differences between these values are not significant and are attributable to the increased precision of the ribl using the ~50-fold larger set of sites. Weakened natural sites, with significantly reduced *R _i_* values that remain above these thresholds, are considered to be leaky (lower affinity binding), whereas those below this threshold are found to completely abolish natural splice site recognition, resulting in either exon skipping or activation of neighbouring cryptic splice sites. However, these outcomes are not mutually exclusive, since leaky splice site mutations may also result in exon skipping and/or activate neighboring cryptic sites. Natural splice sites below these thresholds are extremely rare, and their recognition is likely enhanced through the binding of specific RNA binding proteins that promote exon definition (eg. *XPC* exon 4 acceptor and *MYBP3* exon 12 acceptor ^47, 48^).

Leaky natural sites have *R _i_* values exceeding the *R _i,min_* threshold, which, in theory, retain some capacity to be recognized by the spliceosome. There were 84 variants predicted to cause leaky splicing, of which 19 were shown experimentally to lead to exon skipping without any detectable residual natural splicing ( Supplementary Table 2: #32, 120, 128/380, 195, 276.5, 355, 360, 363, 364, 365, 379, 409, 477/496/934, 573, 842, 853, 883/1589, 886, and 918). Of those, seven are donor splice site mutations at position +5 (Δ *R _i_* ~ -3.5 bits; #128/380, 195, 355, 842, 853, 883/1589, 886), four alter the first exonic nucleotide of a donor site (Δ *R _i_* ~ -3.0 bits; #276.5, 360, 379, 409), and three are donor mutations at position +4 (Δ *R _i_* ~ -2.6 bits; #120, 365, 573). The *R _i,final_* values of these 19 inactivated natural sites range from 2.7 to 8.8 bits, which suggests the possibility that the variant may also simultaneously affect other adjacent or overlapping sites that preclude recognition of the mutated natural site. Additionally, weakening of 11 of these variants activates a neighbouring cryptic splice site, in which no residual natural splicing was detected. However, changes in splice site preference due to small changes in binding affinity within exons are probably related to the processive nature of donor splice site selection ^49^.

Leaky splicing mutations are readily detected when the expressed transcript contains the causative variant or a neighbouring polymorphism. However, there are a number of practical limitations on the methods for experimental validation of leaky splicing mutations. RT-PCR alone would only be considered reliable for confirmation of homozygous mutations (and in one case, a compound heterozygote where two separate variants abolished natural splicing of the same exon), unless combined with a secondary quantitative methodology ^50^. Similarly, it is difficult to assess leaky splicing of heterozygotes using RNAseq data, as reduced levels of wild-type splicing are challenging to determine without adequate read coverage and controls for comparison. However, leaky splicing can be assessed by comparing the frequency of the causative allele to the normal allele in the same cell line when the variant is present within the sequenced reads ^41^. These are special cases however, as the variant itself must either be expressed within an exon or, if intronic, must lead to an activation of a cryptic site further into the corresponding intron.

We previously suggested that weaker splice sites are more susceptible to mutational inactivation relative to stronger sites ^22^. In the present study, all experimentally verified variants affecting natural sites (where leaky and abolished splicing could be differentiated) were analyzed (N = 98). Variants predicted to abolish splicing ( *R _i,final_* < *R _i,min_* and/or Δ *R _i_* < 7.0 bits) were filtered out, as large changes in binding affinity will essentially abolish splicing, despite remaining binding strength and regardless of initial *R _i_* value. Supplementary Figure 1 illustrates the frequency of inactivation by these variants relative to initial *R _i_* value. Variants occurring at weak splice sites ( *R _i,initial_* < 4 bits) abolish splicing in 5 of 6 cases (where Δ *R _i_* < 7 bits), but are not represented as they all weaken the site below *R _i,min_*. The remaining variant slightly weakens a site where R *_i,initial_* is -0.1 bits (where Δ *R _i_* = 0.5 bits), and its recognition may be supported by SR elements ^47^. Moderate strength splice sites (5–11.0) bits are inactivated in 25–60% of cases, and mutations at strong splice sites ( *R _i,initial_* ≥ 12 bits) tend to be leaky ( Supplementary Figure 1b).

Mutations that abolish natural sites (without cryptic splice site activation) are expected to result in a complete loss of normal splicing. However, of the 94 variants that reduced natural splice site strength below *R _i,min_*, 11 were reported to have residual normal splicing activity ( Supplementary Table 2: #185/750, 275, 881, 914, 1315, 1321, 1325, 1326, 1361, 1380, and 1407) ^22, 41, 51, 52^. Two of these occurred at the G of the +1 position of the donor site ( Supplementary Table 2: #185/750 and 1326), which is essentially invariant in functional splice sites. This suggests potential problems in IT or experimental analysis of these mutations. Surprisingly, the majority of these variants occur at the +2 position of a donor splice site and are T>G mutations, which are predicted to abolish splicing activity ^41^. However, the analysis of RNAseq data for these variants showed no splicing defects ( Supplementary Table 7: #1315, 1321, 1325, 1361, 1380 and 1407). One explanation is that resultant aberrantly spliced transcripts were subjected to nonsense-mediated decay (NMD) and degraded. Another possibility is that the coverage of these splice junctions is insufficient to distinguish expression of a single allele from that same allele plus the leaky splice junction. The remaining variants differ in the position within the splice site and decrease natural site strengths to between 0.9 to 2.2 bits ^22, 51^.

Theoretically, a site lacking the canonical G at +1 (donor) or -1 (acceptor) position of a natural site may exceed *R _i,min_*. Ozaltin *et al.* (2011) and Di Leo *et al.* (2009) each assessed mutations at positions +1 or -1, which weaken natural splice sites to *R _i_* > *R _i,min_*, and note that these sites are predicted to be leaky ^53, 54^. However, this is not the sole criterion for interpreting splice site mutations using IT-based methods. The overall change in binding affinity must also be considered, as both mutated sites were predicted to have only 0.4–0.5% of the binding affinity of the corresponding natural splice sites ^53, 54^.

### Branch-point mutations

Although branch-point site (BPS) recognition occurs independently and post-exon definition, mutations in this sequence have also been described, due to its proximity to the natural acceptor site. Following the recognition of and binding to the 5´ss (upstream donor site) by the U1 snRNP, the U2 is recruited to the 3´ss (downstream acceptor) and recognizes the BPS, resulting in the formation of the pre-spliceosome ^55^. Association of U2 with the BPS is essential, as it is the first energy-requiring step, allowing for the tri-snRNP complex of U4/U6 × U5 to be recruited to the BPS, which produces a catalytically active spliceosome ^56^. The BPS typically contains a conserved adenosine and a downstream polypyrimidine tract. It is located within 40 nt of the natural 3´ss, however there are reported cases where it can be up to 400 nt away.

Recognition of the BPS is thus a crucial step in proper splicing, and sequence variants can disrupt this event, impede lariat formation, and intron excision. The complete list of BPS variants analyzed using the ASSA and ASSEDA server can be found in Supplementary Table 5. The variants range in distance from 0–76 nt from the natural acceptor junction, and either weaken, abolish or strengthen the BPS. When validation assays were performed, the prediction by the server was correct in 9/11 cases. We deemed the two other cases to be partially discordant (NM_004628:c.413-24A>G and NM_005902:IVS8-55A>G). ASSEDA predicted these variants to abolish the BPS, but leaky and normal splicing was observed, respectively. The predictions are partially concordant with experimental findings because ASSEDA also predicted the existence of nearby alternative BPS, which if used, could account for the observed phenotype.

**Figure 3.**
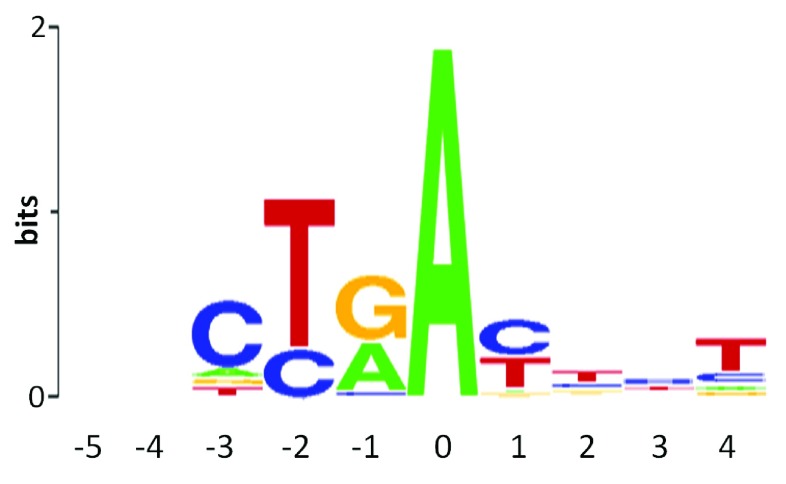
Ribl used for the prediction of a variant’s effect on branch-point sites. Sequence logo for information model for the branch-point site, created using 20 annotated branch-point sequences.

Although IT-based prediction of a variant effects on BPS has been accurate, the number of validated sites used to compute the ribl is substantially smaller (N = 20), and it is not as reliable as those used to determine *R _i_* values of natural acceptor and donor sites. Furthermore, these motifs are short and relatively frequent in unspliced mRNA. One possible explanation for the rarity of BPS mutations is that compensatory, alternative BPS sequences can be recognized and used. Furthermore, the weak constraint on the precision of the distance between the BPS and the 3´ (acceptor) splice site ( Figure 3) further enables activation of these alternative sites. These factors increase the chance that a variant will be falsely predicted to affect a BPS. For example, variants within donor splice site sequences are routinely predicted to alter strength of false BPS. This error is easily avoidable if the potential recognition sequence is filtered for the genomic context of the variant, as well as its proximity to acceptor splice sites.

## Activation of cryptic splicing

**Figure 4.**
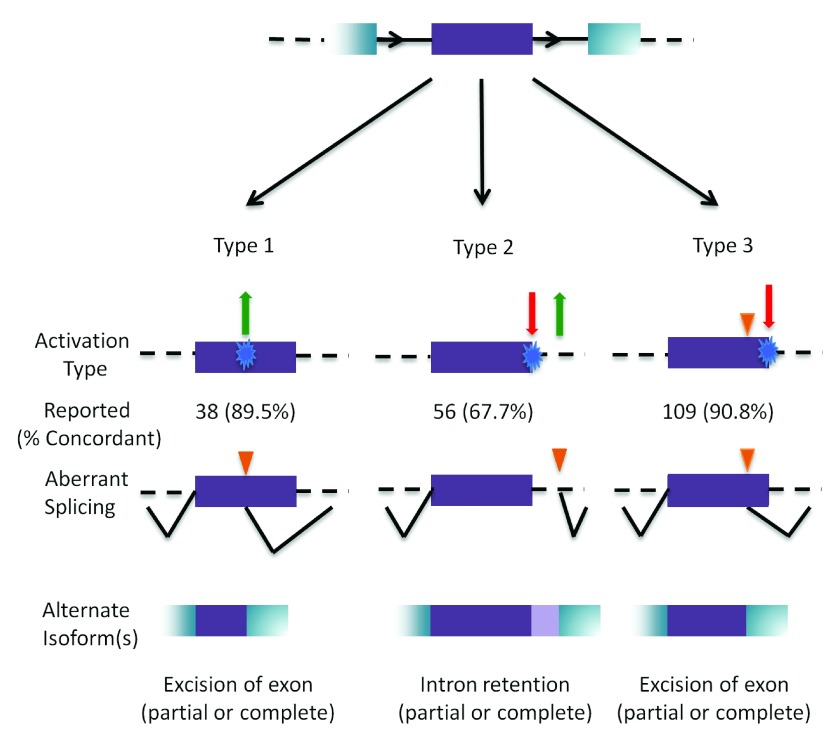
Outcomes of cryptic splicing mutations. A prototypical internal exon (in purple) with flanking exons (in blue); introns are represented by black solid, and dashed lines (top). The three types of cryptic splice site activation are then illustrated. Type 1 cryptic splice site activation (left) is caused by the activation (green arrow) of a cryptic site by strengthening a pre-existing site, or by creating a novel splice site (blue). Type 2 (middle) results from the simultaneous weakening or abolition (red arrow) of the natural splice site while strengthening or creating (green arrow) a cryptic site. Type 3 (right) involves the activation of a pre-existing cryptic site due to the weakening or abolition of the natural splice site (indicated by orange triangle). The number of cases that have been reported in the literature that have been analyzed by IT for each type is indicated, with the percent accuracy in parentheses. The bottom row represents the resulting mRNA structure due to the activated cryptic splice site.

It has been estimated that 1.6% of disease causing missense mutations can affect splicing and recent predictions suggest that approximately 7% of exonic variants in the general population may disrupt splicing, which includes cryptic splicing ^57, 58^. The genome is replete with pseudo (or decoy) splice sites with varying degrees of similarity to natural sites that are not recognized in constitutive splicing ^59^. However, mutations that alter the strengths of either these decoys or the natural splice site of the same polarity may shift the balance of isoforms towards non-constitutive splice isoforms that predominate over or eliminate normal mRNAs ( Figure 4). Mutations can create a cryptic splicing event by creating or strengthening a site in either intronic or exonic regions ( Figure 4, Type 1), weaken the natural site while simultaneously altering an overlapping decoy site ( Figure 4, Type 2), or exclusively weaken the natural site, leading to the activation of a pre-existing decoy site ( Figure 4, Type 3). Although the contributions of cryptic splicing to genetic disease have long been recognized, IT analysis correctly predicts most, but not all, cases ( Figure 4). The challenges in identifying potential cryptic sites or determining activation are attributable to our incomplete understanding of the requirements for activation ^60– 62^, which include exon length, processivity of donor site recognition, and involvement of splicing regulatory factors. A database of aberrant 3´ and 5´ splice sites has been compiled ^62^.

**Figure 5.**
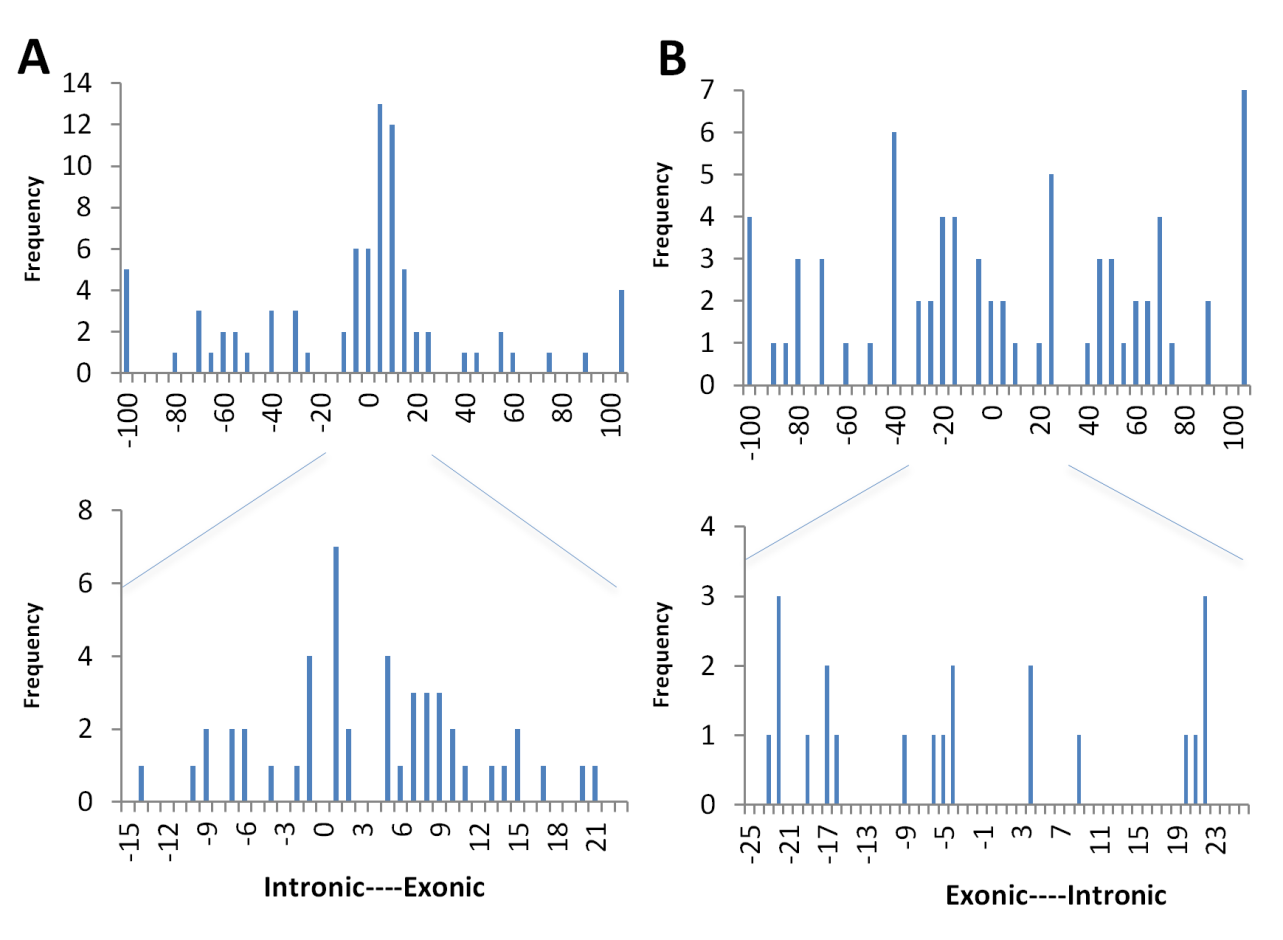
Distribution of activated cryptic sites. The frequency of validated cryptic splice acceptors ( **A**) and donors ( **B**) occurring at positions relative to the natural splice site. Positions are given using ASSEDA coordinates. Lower panel expands the cryptic site distribution of the region circumscribing the natural splice site.

Another bioinformatic method for cryptic site recognition relies on a training set composed of cryptic sites that are known to be used ^63^. There are a number of drawbacks to this approach: the training set is itself not representative of all cryptic sites; and sites that are altered but unused cannot be discriminated from those that are activated (since the latter group also depends on the strength of the corresponding natural splice site). IT-based methods rank cryptic and cognate natural site strength in a way that predicts whether the site will be activated, as well as the abundance of each pair of splice isoforms. Furthermore, the structures of the prospective isoforms are presented by ASSEDA with relative quantitation of each, both prior to and post-mutation.

During our review, we noted 203 variants with experimental support for cryptic splicing ( Supplementary Tables 6–8). Of these, 38 variants resulted in Type 1 cryptic splicing. From those, site activation (existence of the site and strength ≥ 2.4 bits ^22^) was correctly predicted by ASSEDA in 34 cases (89.5%). We identified 56 variants resulting in Type 2 splicing, 38 of which (67.7%) were accurately predicted, while the remaining 119 variants resulted in Type 3 cryptic splicing and 99 (90.8%) of the alternate splice sites matched predictions.

Prediction of Type 3 cryptic splicing was more accurate than Types 1 or 2. The criteria for concordance with experimental data were that ASSEDA predicted both the cryptic site and that the variant weakened the natural site. However, the strength of a site is not the sole determinant of whether or not a site is activated. Unlike natural sites, novel cryptic sites are not under selection to maintain binding to the spliceosome, and their genomic context is less constrained than natural splice sites. The presence of cooperative splicing enhancer or repressor elements adjacent to cryptic sites, which could influence cryptic splice site activation, is not yet predictable. Additionally, many of the reported activated cryptic sites have been confirmed using non-quantitative approaches, and these may not constitute the predominant splice forms relative to constitutive exons with stronger natural sites. Finally, certain isoforms may not be detected; as aberrant transcripts are often subject to degradation and the tools used to evaluate functional splicing consequences do not always have sufficient resolution to distinguish small differences in isoform structure. All of these factors can affect the concordance of predicted cryptic site activation with experimental validation.

We also separated each sub-group of cryptic splice variants by location (intronic vs. exonic) and computed the average difference in strength between pairs of natural (post-mutation) and the activated cryptic sites. For intronic Type 1 variants, activated cryptic sites were 0.86 ± 5.28 bits stronger than the corresponding natural site (N = 12). There were eight Type 1 variants (4 at acceptors and 4 at donors) that were missed, because the *R _i,final_* value of the natural site exceeded the strength of the corresponding cryptic site by ≥ 1.0 bits (variants with Δ *R _i_* < 1.0 bits are not reliably detected experimentally). We hypothesize that these cases could be explained by concomitant changes in surrounding regulatory binding site sequences. Exonic Type 1 variants were often slightly weaker than their cognate natural sites (-1.1 ± 3.8 bits; N = 26). Nearly all of these involved ectopic donor site activation (12 of 13), consistent with a processive mechanism for donor site recognition, which searches downstream from the acceptor splice site to the first donor site of sufficient strength to form an exon ^35^. The opposite pattern was observed with intronic Type 2 cases, in which 20 of 21 exceptions occurred at acceptor sites. On average, the activated cryptic site exceeded the strength of the cognate natural site (1.3 ± 4.6 bits; N = 57). Activated, exonic Type 2 acceptor cryptic sites tended to be weaker than their natural site counterparts (-2.2 ± 3.3 bits; N = 4). This result may be attributable a low sample size, with 2 of these mutations exhibiting natural sites that were stronger (≥ 1.0 bits) than the corresponding cryptic site (1 donor and 1 acceptor). Finally, Type 3 activated intronic cryptic sites exhibited the greatest difference between the strengths of cryptic sites and cognate natural splice sites (6.3 ± 4.9 bits; N = 104). This category contained the fewest number of exceptional cryptic sites, with *R _i_* values less than those of natural sites (5 acceptors and 3 donors). This is consistent with the idea that the intronic cryptic sites are generally not under selection for adjacent functional regulatory binding sites, and, in order to be activated, are required to be substantially stronger than the natural site. Although *R _i,final_* values were stronger (2.1 ± 1.9 bits; N = 20) than the natural site, exonic Type 3 cryptic splice sites did not show as great a difference in strength with a single exceptional case (of an acceptor). Despite these exceptions, activated cryptic splice sites are generally stronger than the corresponding natural splice sites ^22^.

## Combinatorial effects

While functional natural splice sites and an intact BPS are integral for accurate and efficient splicing, other genetic elements have been shown to make essential contributions to exon definition ^64^. Introns will often contain more than one potential splice site recognition sequence, but nevertheless, the correct natural site is consistently selected ^59^. Differences among the strengths of potential sites, as determined by IT analysis, are a major, but not the sole, determinant of splice site utilization. The implication is that additional sequences within the gene are necessary to ensure specificity and precision of exon recognition. Studies of facultatively expressed alternative exon structures have revealed *cis*-acting sequence elements that function to enhance or repress exon recognition. These sequences cooperate with factors that recognize natural splice sites, whose sequences and relative strengths can vary considerably. Depending on their context, these elements have been referred to as exonic splicing enhancers (ESEs), exonic splicing silencers (ESSs), intronic splicing enhancers (ISEs) or intronic splicing silencers (ISSs). In general, these elements serve as binding sites for *trans*-acting elements, which will either promote or impede the spliceosomal recognition of a splice site. The majority of enhancer elements will act through the recruitment of SR proteins and associate components of the U1 and U2 spliceosomes ^65, 66^. Silencers are often of the hnRNP class, which act through a diversity of mechanisms including steric hindrance, the formation of dysfunctional complexes, or blocking processiveness ^67– 69^. To add to the complexity of splicing regulation, it has recently been shown that SR protein function is dependent on context, i.e. whether the corresponding binding site is intronic or exonic ^70, 71^.

To improve accuracy of exon definition, the strengths of regulatory elements (i.e. their *R _i_* values) have been incorporated into splicing mutation prediction. The significance of regulatory elements in disease has been demonstrated in many cases. For example, in the *NF1* gene, ESE disruption is the primary cause of exon skipping ^72^. Many other genes, including *APC*, *SMN*, *BEST1, PDHA1* ^73– 76^ have been proven to harbour variants that disrupt ESEs and have a confirmed impact on mRNA splicing.

Adding to the complexity, the recognition sequences for these RNA binding factors, while well defined, tend to be short, and can vary to the degree that the same sequence may contain overlapping elements of binding sites for multiple factors. However, this does not necessarily imply that such a sequence is bound with similar affinity by each factor or that it contributes to exon definition. At the same time, these sequences tend to be evolutionarily conserved and may be required for proper splicing ^77, 78^.

ASSEDA optionally incorporates PWMs for regulatory binding sites for mutation analysis ( Table 1) in addition to the default donor and acceptor sites. The program selects the most proximate predicted ESE/ISS to the natural splice site when calculating *R _i,total_*. The molecular phenotype, which dictates the splice isoforms that are predicted and their relative abundance, accounts for both the potential effect on the natural site and the most relevant splicing regulatory site. For these regulatory binding sites, a second gap surprisal term specific to the ESE/ISS of interest is applied to the *R _i,total_* calculation ^35^. The gap surprisal functions for SF2/ASF and SC35 have been previously described ^35^, where the most common distance of the ESE/ISS is within 10nt of the natural site. The gap surprisal penalty gradually increases with distance from the natural site. Gap surprisal distributions for ELAVL1, TIA1 and SRp55 show a similar pattern, while hnRNPA1 and PTB binding sites are strongly clustered around splice junctions. It should be feasible to include the contributions of multiple splicing regulatory binding sites of the same or different RNA binding proteins in determining *R _i,total_*; however this capability had not yet been implemented. Currently, if multiple sites of the same type are altered, the strongest (before or after mutation) is chosen by ASSEDA software.

**Table 1.** Splicing regulatory protein binding sites ASSEDA scans for and their associated effect on
splicing.

Splicing Factor	*R _sequence_* (bits)	Sequence Logo	Location-dependent effect on splicing	
			Intronic	Exonic
hnRNPH1	8.9 ± 1.8	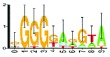	E ^79, 80^ / S ^81, 82^	**S** / E ^83^
hnRNPA1 ^i^	4.6 ± 1.5	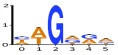	**S** / E ^84^	**S**
TIA1	7.6 ± 3.1	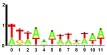	**E**	N/A
SRSF6 (SRp55)	5.2 ± 1.4	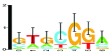	E / S ^82^	**E** / S
SRSF5 (SRp40)	4.5 ± 1.5	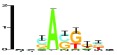	E / S ^82^	**E** / S ^85^
SRSF2 (SC35)	4.5 ± 1.6	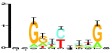	E / S ^86^	**E** / S ^87^
SRSF1 (SF2/ASF) ^88^	5.8 ± 1.5	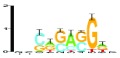	E / S ^86, 89, 90^	**E** / S
PTB ^ii^	4.9 ± 1.9	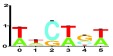	**S** / E ^91^	**S**
ELAV1	9.6 ± 3.4	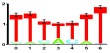	S / E / N ^92, 93^	**S**

Reported dominant effect is bolded. E – Enhancer; S – Silencer; N - Neutral.

^i^Enhancer activity by hnRNP A1 occurs at the junction ^84^. ^ii^PTB does not directly enhance splicing, but can do so indirectly by preventing the binding of splicing repressors ^91^.

Although the disruption of splicing regulatory sequences can cause aberrant splicing, the interpretation of variants affecting these sites is not as straightforward. Due to their degenerate nature, short sequence, and a lack of understanding of the context of their use, altered regulatory sites should be functionally validated before being deemed pathogenic ^7^. Using variants from a number of different studies, ASSEDA accurately predicted experimentally determined changes in binding at a splicing regulatory site 75% of the time (N = 12) ^35^. However, there were instances where regulatory sequences had been analyzed by IT, and considered to contribute to disease, but the results were not reproducible. For example, Kölsch *et al.* (2009) described SNPs associated with Alzheimer’s Disease, one of which strengthened and created SRp40 and SRp55 sites, respectively, but were reported by authors to be abolished ^94^. This study did not report any evidence to support the significance of these predictions.

Functional validation of the effects of these mutations could contribute to understanding the roles of these factors in regulating constitutive splicing. Similarly, there is still little understanding on how multiple regulatory binding sites within the same region function as a unit. Using a pull-down assay, Olsen *et al.* (2014) demonstrated how different variants affect the binding of multiple regulatory proteins. One mutation was predicted to create and strengthen multiple hnRNPA1 sites and slightly strengthen an SF2/ASF (SRSF1) site. The pull-down studies showed up-regulation of hnRNPA1 binding and a decrease in SF2/ASF binding. However, SF2/ASF binding increased when a mutation disrupting hnRNPA1 affinity was introduced, suggesting that the strong hnRNPA1 sites outcompete the weaker SF2/ASF site.

In some instances, alterations in regulatory splice site recognition sequence and natural splice strength occurred concomitantly, with both predicted to have similar effects on splicing. Alteration of a regulatory sequence can sometimes provide a plausible explanation for discordant *in silico* prediction and experimental validation. As an example, Smaoui *et al.* (2004) analyzed a donor site mutation (NM_001040667:c.1327+4A>G) in *HSF4* in a family with congenital cataracts ^50^. This variant was predicted to cause leaky splicing ( *R _i,final_* = 5.4 bits; Δ *R _i_* = -2.6 bits; 67.5% residual binding), however RT-PCR showed complete exon skipping. Our further analysis showed that it is predicted to also create an overlapping hnRNPA1 site ( *R _i,final_* = 4.2 bits; Δ *R _i_* = 17.1 bits). Another case involved a mutation in the *XPC* gene (NM_004628:c.2033+2T>G) that created a novel intronic cryptic site 4 nt downstream of a natural donor site ^95^. However, a weaker site 68 nt downstream from the natural site was activated. A possible explanation could be that activation of the cryptic site is influenced by a neighbouring hnRNPA1 site that is itself strengthened ( *R _i,final_* = 5.2 bits; Δ *R _i_* = 2.2 bits) and an SRp55 site that is significantly weakened ( *R _i,final_* = 1.9 bits; Δ *R _i_* = -4.0 bits).

The effects of changes in regulatory binding site strengths may ascribe potential functions to previous VUS. For example, Maruszak *et al.* (2009) present a *PIN1* variant associated with late-onset Alzheimer’s Disease (NM_006221:c.58+64C>T) ^96^. Based on IT, it is expected to abolish an intronic SC35 site, which could have either an enhancing or silencing effect ( Table 1). A 2.82-fold decrease in transcript levels was demonstrated, which is concordant with previous findings reporting decreased *PIN1* levels in the brains of Alzheimer’s Disease patients. Another study described an exonic missense variant within the *ETFDH* gene in a patient with multiple acyl-CoA dehydrogenase deficiency (NM_004453:c.158A>G) that showed evidence of exon skipping. The variant was predicted to be “benign” or “tolerated” when evaluated with PolyPhen and SIFT ^23^. ASSEDA, on the other hand, predicted the creation of an hnRNPA1 site ( *R _i,final_* = 5.9 bits; Δ *R _i_* = 17.1 bits), a slightly strengthened hnRNPH site ( *R _i,final_* = 4.0 bits; Δ *R _i_* = 0.2 bits), the abolition of an SRp40 site ( *R _i,final_* = -3.3 bits; Δ *R _i_* = -6.3 bits) and two novel, weak SF2/ASF sites ( *R _i_*_,final_ = -4.6 bits; Δ *R _i_* = 0.8 bits and *R _i,final_* = -2.4 bits; Δ *R _i_* = 0.4 bits) ^23^. The natural donor site was unaltered by the mutation. As indicated earlier, the mutation was confirmed experimentally to increase hnRNPH and hnRNPA1 and decrease SRp40 and SF2/ASF binding.

## Validation of results

A number of early mutation studies did not perform expression analysis and relied solely on the ASSEDA or ASSA server to interpret potential mutations. This is not recommended, as there are limitations to any *in silico* predictive method, which impacts accuracy and precision of the prediction. Assuming that the impact of the mutation on expression can be detected, experimental validation of IT-based mutation analysis can reveal its limitations. We describe the various validation methods that were employed in the articles where expression data were available. Below, advantages and disadvantages of these approaches are explored, as well as how lower sensitivity validation can result in misinterpretation. Finally, we determine the accuracy of IT-based prediction, and point out some instructive, discordant cases.

### Validation methods

The two most widely used methods for validating mutant mRNA splicing isoforms have been RT-PCR analysis of patient mRNA, and transfection of minigene constructs expressing the mutated exon into cell lines, followed by RT-PCR. These assays were, in some cases, accompanied by other techniques such as direct sequencing of cDNA, Western blotting, luciferase expression assays or immunostaining. A number of studies used quantitative RT-PCR or real-time PCR to estimate isoform abundance. RNA or cDNA sequencing and exon expression microarrays were also used in several studies to support *in silico* predictions. Certain functional assays that we reviewed were unique to a single study, including: allelic instability, exon trapping, immunoprecipitation of splicing factors, and flow cytometry ^23, 97– 99^. Other indirect methods of justifying the association between a splice site variant and disease included fundoscopy, loss of heterozygosity, blood protein levels, and segregation with disease ^100– 103^. Because a variant may result in aberrant splicing but might not be accompanied by a detectable phenotypic change, we excluded the results of indirect assays of phenotype. Indirect measures of phenotype can support disease association, but do not inform about accuracy of splicing prediction.

Endpoint RT-PCR and minigene assays probe the specific variant in question, but do not reveal relative abundance of each isoform, whereas qPCR does. Neither method resolves mRNA sequence at the nucleotide level, which can fail to confirm predicted splicing mutations, especially in instances where a small number of nucleotides are retained at the constitutive splice junction ^104^. The resultant frameshifted mRNAs can cause premature truncation of the transcript (PTC), instability, and NMD, leaving no evidence of the mutated isoform (unless the cells had been treated with an NMD inhibitor). A disadvantage is that in cases where the protein is not degraded, but still impaired or dysfunctional, the result will be incorrectly categorized as benign. For example, Wessagowit *et al.* (2005) used sequencing of a *COL7A1* variant (NM_000094:c.341G>T) to demonstrate a 87 nt deletion in the cDNA ^105^. The authors also performed immunostaining of the corresponding protein with a monoclonal antibody, which showed no difference between wild-type and mutant samples because the epitope was not disrupted by the deletion. Had the authors only performed the binding assay, the variant would have likely been disregarded. NMD can be a predominant cause of false-negative results when validating splice variants. When aberrant splicing causes a frameshift and PTC, translation of truncated proteins is prevented, which otherwise can have dominant negative effects or exhibit gain-of-function ^106^. However, if these transcripts are degraded and only the normal allele is detectable (in the case of a heterozygote or leaky splicing), then the splicing prediction will not be supported. Interestingly, Khan *et al.* (2004) were able to show that NMD had occurred by comparing levels of total message (qPCR) between wild-type and mutant samples ^47^. Experimental methods have been developed to stabilize transcripts with premature termination of translation, thus circumventing NMD. The use of emetine, which inhibits translation and stabilizes RNA transcripts, can increase the relative amount of aberrant transcript observed ^107, 108^. However this approach can induce a stress response within the cell and further transcription must be halted using actinomycin D. This combination was used by Bloethner *et al.* (2008) in an approach called Gene Identification by NMD Inhibition ^109^. Similarly, the use of puromycin and cycloheximide were shown to inhibit NMD and restore predicted aberrant splice forms ^97, 110^. Furthermore, certain mutations proximate to the penultimate exon evade NMD ^111, 112^.

### Regulatory sequence variants

A number of assays have been developed to confirm direct effects of variants on splice site recognition, however fewer methods are available to measure effects of mutations at binding sites of splicing regulatory proteins ^113^. The most reliable approach is to associate a change in splicing with a change in regulatory protein binding. A combination of electrophoretic mobility shift assay and RT-PCR were used to confirm that a predicted change in an SF2/ASF binding site caused exon skipping in the *CFTR* gene ^114^. Others performed RNA affinity purification in combination with Western blotting ^23^.

Another approach tests multiple variants at the same position through minigene assays. Anczuków *et al.* (2008) observed that two variants at the same position in *BRCA1* (c.3600G>T and c.3600G>C) predicted different effects on regulatory sequences, as well as different observed effects on splicing ^115^. The G>T variant predicted abolition of a SRp40 site and weakening of an SF2/ASF site by both ASSA and ESEfinder, and showed a significant reduction in the relative amount of normal transcript. The G>C variant, which did not elicit a change in splicing, was not predicted by ASSA to have a significant effect on either site (although ESEfinder predicted weakening of the SRp40 site below its default threshold). The difference in splicing efficiency could be due to the loss of binding by one or both of these regulatory proteins. This assay associates predicted changes to regulatory protein binding site strength to changes in splicing. A direct binding assay would lend key support for such predictions.

## Accuracy of IT-based prediction

We previously evaluated the accuracy of IT-based prediction using a set of validated splicing mutations (85.2%; N = 61) ^25^. Other studies have also evaluated the accuracy of ASSA/ASSEDA while evaluating differences between multiple predictive programs and have shown varying levels of concordance (68.8%, N = 16; 90.1%, N = 22; 100%, N = 24) ^51, 104, 116^. With a comprehensive list of all published variants analyzed using IT-based methods ( Supplementary Bibliography), we perform a meta-analysis of all of these variants to minimize bias in interpretation and impact of ascertainment of specific phenotypes from individual studies. The list of variants is more extensive than any previous study examining accuracy of IT-based methods. The variants are not restricted to a single or even group of diseases, but rather cover over 150 different conditions (see Supplementary Table 2).

In total, 905 variants were reported in 122 different publications to have been validated for their effects on splicing (1,727 total variants analyzed from 216 papers – Supplementary Table 9). In all cases, the authors performed information analysis; however, the validation experiments were sometimes contained in the original reports and in other cases, later studies. In a minority of mutations, the validation results were either uninformative (N = 36) or the methods did not directly imply an effect on splicing (N = 2); these mutations were therefore excluded in determining the accuracy of predictions (shaded in grey in Supplementary Table 9).

More specifically, in order for experimental results and predictions to be considered concordant, one or more of the following criteria had to be met:

a. A variant predicted to abolish a splice site did abolish splicing, with no residual splicing observed. Exceptions to this were assays in which both the mutant and wild-type alleles were expressed in the same cell line or patient sample, and could not be discriminated from one another (i.e. RT-PCR);
b. A variant predicted to be leaky exhibited residual normal splicing, with the exception of cases where a much stronger cryptic splice site was activated;
c. A variant that strengthened the natural site and showed normal or increased levels of the wild-type isoform, consistent with it having a benign phenotype and/or polymorphic;
d. A variant predicted to activate a pre-existing splice site, while also reducing the natural splice site strength, was demonstrated experimentally to result in cryptic splicing, regardless of whether it was predicted it to be the predominant isoform;
e. A variant predicted to affect a splicing regulatory protein-binding site was consistent with validation experiments explicitly assessing binding affinity and associated splicing alterations.

Cumulatively, 87.9% of variants documented by expression studies (762 of 867) that satisfied these criteria were accurately predicted by ASSEDA. A minority of papers reported variants to be “partially concordant” (3.1%; 27/867), meaning that while the cryptic site observed was predicted, it was not the most likely splice isoform relative to other expressed cryptic exons. Because this method of scoring met our criteria (see point d above), we included these in our determination.

### Predicted mutations discordant with validation results

Limitations of both the predictive model and the validation data/methods were the primary reasons for discordance. Where information analysis predicted a neutral change or no effect, but validation showed aberrant splicing, we hypothesize that there are either unrecognized splicing regulatory protein binding sites that are weakened or abolished, or that there are underlying mechanisms that are not currently addressed by current information models ^23, 35, 50– 52, 54, 96, 98, 99, 114, 117– 127^. The validation methods used can also contribute to discordant results. We note that 41 discordant results originated from one of our own studies ^41^. This study used RNAseq data to validate predictions, a genome-wide approach that should be used with caution when inferring changes resulting from potential splicing mutations. Until this study was published, IT-based mutation analysis was based on single or candidate disease gene studies. RNAseq reveals all changes in transcript levels for all genes, which although potentially relevant to splicing, may not necessarily contribute to the phenotype in question. This leads to the possibility, especially in cancer phenotypes, of bystander effects (global splicing dysregulation, natural alternative splicing) that are not directly attributable to the predicted mutations. Furthermore, because the sequence reads at splice junctions are short and often limited in number, a relevant splicing aberration may result from a given variant, but it was not detectable. Finally, the predictions of IT can pick up variants that should alter splicing for example, of rare recessive alleles, that that may not have any disease relevance.

## Misinterpretation of variant effects

While preparing this review, several variants misinterpreted with IT-based tools were noted. These variants have been re-analyzed to disseminate the correct findings and to avoid making similar errors in the analysis of newly discovered variants. Supplementary Table 2 contains these results. The most common problems result from unfounded emphases on altered or pre-existing cryptic sites that are determined to be significantly weaker relative to the cognate natural site ^109, 128– 132^, and from selectively reporting a single change in the *R _i_* value when, in fact, multiple significant changes can be detected ^48, 128, 133– 136^. An example of the first type of error is exemplified by a variant in *CGI-58 (ABDH5*), where the natural splice site is 9.1 bits (or ≥ 549-fold) stronger than the reported cryptic site ^129^. Henneman *et al.* (2008) selectively reported the effect of a mutation that weakens a natural donor splice site in *APOA5*, however only a change in the information content of an SC35 binding site was indicated.

Other common problems include incorrect declaration of small Δ *R _i_* values as significant changes ^109, 137, 138^, use of incorrect *R _i,min_* values ^139, 140^, and the computation of predicted binding strength changes on a linear scale ^141^ rather than the correct exponential function (i.e. ≤ 2 ^Δ *Ri*^) ^18^. Smaoui *et al*. (2004) described an 8.0 bit donor site as weak, which is actually equivalent in strength to *R _sequence_*, the average strength ^33^. Allikmets *et al.* (1998) and Ozaltin *et al.* (2011) both described an inactivating mutation as leaky, because the weakened site remained above the *R _i,min_*. However, the variant mutation produces a site with < 0.7% of its original binding affinity, which would substantially reduce exon recognition and lead to exon skipping ^116^. Also, cryptic sites created in the promoter regions of genes should not be considered to be splicing mutations ^142^. Variants that are predicted to create a cryptic site upstream or overlapping a natural site of the opposite polarity (i.e. cryptic donor upstream of a natural acceptor) have been reported ^131, 132, 143^, which would be inconsistent with established splicing mechanisms ^35^. A rare exception that could render such a site active is to the creation of a cryptic exon that occurs in conjunction with a proximate, correctly oriented, pre-existing cryptic splice site of opposite polarity ^22, 30^. Insufficient numbers of examples of mutations creating cryptic exons have been reported to date for ASSEDA to accurately predict these exons by default.

Several results were generated by incorrect entry of mutations in to ASSA/ASSEDA. For example, altered cryptic splice sites have been confused with natural sites ^48, 137, 144, 145^. Additionally, ‘residual binding strength’ displayed has been misinterpreted as a percent decrease ^144, 146^. Strong, pre-existing cryptic sites outside of the default sequence analysis window (54 nt circumscribing the mutation) have also been missed because the window was not expanded to include these sites ^147^. Although the predicted isoform structure generated by ASSEDA will, by default, display skipping for mutated natural sites with Δ *R _i_* ≥ -7.0 bits (or ≥ 128-fold) ^116^, smaller decreases in natural site strength of an internal exon can sometimes partially induce exon skipping. This value is adjustable, and it may be advisable to explore different thresholds depending on the particular susceptibility of a splice junction to exon skipping. Sharma *et al.* (2014) used the default threshold from ASSEDA to interpret *CFTR* mutations c.2988G>A (9.6 to 6.6 bits, natural donor site of exon 18) and c.2657+5G>A (9.1 to 5.6 bits, natural donor site of exon 16), but exon skipping was documented. IT analysis was not discordant for these variants, which significantly weaken the corresponding splice sites by ≥8- and 11-fold, respectively, and has been shown in other genes to lead to exon skipping, leaky splicing, or both of these outcomes. Aissat *et al.* (2013) tabulated variants that were predicted to affect strengths of ESE binding sites, but did not comprehensively report all findings even though predictions by ASSA and ESEfinder were concordant (eg. *CFTR*: c.1694A>G). Alternate mutation entry methods, which do not use contextual gene name annotations, such as entry by rsID, report predicted binding changes on both strands. A report indicating abolition of SRp40 binding sites on the antisense strand was confused with binding sites for *CYP46A1,* which is transcribed from the sense strand ^148^.

Other problems include inadvertent mislabelling of splice site type or location ^149– 151^, interchange of the terms information content and change in information ( *R _i_* and Δ *R _i_*) ^122^, and unclear variant interpretation (i.e. “run on into the intron”) ^152^. Moriwaki *et al.* (2009) claim ASSA did not predict a mutated natural donor site, but in fact, the site was present in our reanalysis ^153^. Published *R _i_* values from Rogan *et al.* (1998) and von Kodolitsch *et al.* (1999) are in some instances different from current values due to updates of the reference genome sequence. Nevertheless, the overall predicted effect did not change, but initial and final *R _i_* values were inconsistent. Interpretations of certain mutations could not be reproduced in some instances ^103, 145, 154– 156^. Finally, we noted that ASSEDA can sometimes improperly parse indels entered using c. or IVS notation. Such errors have led to published false results ^67, 116, 157, 158^.

## Interpretation of published variants in studies that use information analysis

### Genotype-phenotype association

The severity of phenotype due to splicing mutations can be related to their effects on mRNA splicing, after careful consideration of the overall impact on mRNA levels and protein coding ^159^. Significant information changes (where Δ *R _i_* ≥ 7.0 bits or where *R _i_* ≤ 2.4 bits) of splicing variants in hemophilia patients ( *F8C* and *F9*) were shown to correspond to the severe clinical phenotypes of the disease (reduced protein activity, increased clotting time, bleeding frequency) ^127^. The overall effect on the coding potential of the mutated transcript should be considered, as skipping events that maintain the reading frame commonly lead to milder phenotypes ^160– 162^. Nevertheless, two variants that abolish splice site recognition in *PTPRO* in Idiopathic Nephritic Syndrome reported by Ozaltin *et al.* (2011) had similar phenotypes even though one retained the reading frame and the other caused a frameshift. The exon deleted by the in-frame skipping event is highly conserved ^53^. Exon skipping events that cause frameshifts close to the carboxy-terminus may lead to mild phenotypes, as they avoid NMD ^112– 163^. Dominant negative mutations with either *R _i_* > *R _i,min_* or with modest decreases in Δ *R _i_*, may be less likely to cause severe phenotypes, as a residual amount of the natural isoform continues to be expressed ^103, 117, 141, 164– 168^. The impact of cryptic site-activating variants on phenotype can be similarly assessed. Activated cryptic sites which shift the reading frame have been shown to be more severe clinically relative to those which maintain the same reading frame as the native gene ^105, 162, 169, 170^.

IT-based tools exhibit high specificity for analysis of splicing neutral variants in hereditary breast/ovarian cancer and other disorders ^116^. These predictions can reduce the requirement for experimental validation of low-priority candidate mutations with minimal changes in information content ^14, 22^. IT analysis has been used in numerous studies to infer neutral effects of variants ^14, 34, 97, 109, 116, 119, 128, 129, 151, 156, 157, 171– 185^. Similarly, variants that strengthen natural splice sites ^186– 188^ are also likely to be neutral, though these variants can increase retention of exons that are otherwise frequently alternatively spliced ^189, 190^. However, binding site variants with minimal splicing information changes may still alter mRNA processing by disrupting mRNA secondary structure ^191^.

## Polymorphisms and splicing

Early studies suggested that common polymorphic sequence variations at splice sites corresponded to small Δ *R _i_* values, consistent with these changes having little impact on mRNA abundance ^22^. More recently, it has been appreciated that certain rare SNPs have significant genetic loads, can actively alter mRNA splicing profiles, and lead to non-obvious splicing phenotypes ^58, 189^. Nevertheless, it is not uncommon for reports to solely analyze novel variants and ignore known SNPs ^136, 156, 158, 192^, or limit results only to those that occur in the vicinity of natural splice sites ^184^. We find that 56.4% of common SNPs (with population frequencies ≥ 1% in Supplementary Table 2) within natural sites significantly alter their strength (12.8% abolish and 28.2% cause leaky splicing, 15.4% modestly strengthen sites [Δ *R _i_* < 2.6 bits]), and 43.6% have insignificant Δ *R _i_* values, as expected (N = 39). The mean *R _i,final_* and Δ *R _i_* values, for these natural sites are 7.9 ± 4.0 bits and -1.4 ± 3.0 bits, respectively, which suggests the effects of these polymorphisms on splicing are nil to limited. However, polymorphisms can significantly modulate splicing, as some common SNPs are predicted to abolish natural splicing ( Supplementary Table 2: #1291, 1296, 1431, 1435, and 1436). These include rs10190751 in *CFLAR*, which modulates the production of two short isoforms, and is associated with an increased risk of lymphoma ^189, 193^, rs3892097, which alters exon inclusion in *CYP2D6* ^30^ and leads to a non-functional protein and altered drug metabolism ^194^, and rs1805377 in *XRCC4* ^34^, which has been associated with oral cancer susceptibility ^195^ and increased risk of gliomas ^196^. There is also experimental support for common SNPs that have been predicted to affect splicing ^22, 98, 107, 110, 114, 118, 149, 164, 189, 197^. For example, experimental evidence for increased exon inclusion has been described for three of six SNPs that increase strength of natural splice sites ^189, 190^. Numerous common SNPs, which were either deemed neutral or predicted to affect splicing, have not been confirmed experimentally ^14, 22, 25, 34, 52, 94, 99, 131, 133, 144, 145, 148, 151, 165, 166, 168, 179, 183, 185, 188, 198– 208^. Polymorphisms with significant information changes should be investigated, as they may not be completely benign and can have a significant impact on mRNA splicing.

## Inference of variant pathogenicity by IT analysis

Recently, American College of Medical Genetics and Genomics recommendations for reporting incidental findings in sequencing have suggested that bioinformatic predictions are not sufficient to declare clinical significance ^209^. Preceding the publication of these guidelines, numerous peer-reviewed articles suggested variants analyzed by IT to be causative/pathogenic/disease-causing, without confirmation of the predicted splicing effect ^101, 135, 137, 150, 160, 167, 178, 205, 210– 218^. Other authors have qualified the interpretation of bioinformatic results with less certain terms (i.e. ‘suggest’ and ‘likely’ pathogenic) ^110, 112, 176, 219– 223^. Leclerc *et al.* (2002) ^165^ state that a predicted variant confirmed to affect splicing is likely deleterious, but could not be unequivocally shown to cause the observed phenotype. Although IT predictions can relate a sequence change to the resultant phenotype, caution should be exercised when deeming a predicted splicing variant as pathogenic in the absence of other functional evidence. The high level of concordance between IT mutation analysis and experimental findings indicates that this approach, in conjunction with other evidence, can be used to detect splicing effects, which may be used to explain disease phenotypes.

## Comparisons to other software programs

There are now over a dozen other publically available splicing prediction tools, some using strategies similar (MaxEntScan [MES]) and others, which are quite different (NNsplice) that are compared with IT ^224, 225^. Vreeswijk *et al.* (2009) assessed the applicability of different splice prediction programs to diagnose *BRCA1/2* variants. These authors recommended that the outcome of 3 programs was sufficient for analysis, unless all three predictions were discordant from one another (2 for false positive predictions). Despite the obvious appeal of consensus between different analytical methods, a major caveat in using polling strategies for mutation assessment is that these approaches are prone to both systematic and sampling errors ^40^.

We summarize results of 36 publications that used both IT-based prediction tools and one or more alternate prediction tool (14 for 5´ and 3´ splicing, six for splicing regulatory proteins) to assess mutations ^23, 39, 97, 99, 103, 111, 114– 117, 123, 130, 132, 141, 147, 156, 158, 165, 166, 171, 179, 185, 189, 197, 210, 218, 226– 235^. The analysis performed by the authors allowed us to compare the similarity of predictions to those programs and IT in Table 2a and Table 2b. Those most commonly used for 5´ and 3´ splice sites (NNsplice, MES, NG2, HSF and SSF) were highly concordant for natural sites (85.4% for donor and 77.6% for acceptor sites; Table 2a). Discordance of acceptor predictions may be due to methodologies that do not analyze the complete acceptor site (HSF analyzes only 14 intronic nucleotides upstream of acceptor splice sites) ^236^. Some programs (SSF, HSF) exhibit greater concordance with IT for cryptic splice site prediction (96% for donor and 76.9% for acceptor sites). The level of discordance between IT and other commonly used software programs (59.5% for donor and 60% for acceptor sites) may be attributable to the empirically-derived scoring thresholds and the validation sets used to predict mutated splice sites. Models that are typically built (or trained) using known natural splice sites may be less sensitive for differentiating true cryptic splice forms from decoys in the genome, which tend to be weaker than natural splice sites. Tools are highly consistent when analyzing variants expected to be neutral with respect to splicing (100%; N = 71). Colombo *et al.* (2013) compared nine programs to evaluate accuracy in predicting mRNA splicing effects and reported that ASSA, along with HSF, demonstrated 100% informativeness and specificity.

ASSEDA has also been used to analyze RNA binding proteins that enhance or silence exon recognition ( Table 2b). ESEfinder was used for 42.2% of these mutations in one or more regulatory binding sites ^237, 238^. However, variants predicted by ESEfinder to have deleterious effects are discordant with some IT predictions (6 of 15; Table 2b). The discordance with ESEfinder may be associated with differences in the respective analytic methods, as several of the models (SF2/ASF, SC35, SRp40) used by ASSA and ESEfinder were created from the same source of experimental data ^87, 239^. While the majority of the discordant results were cited in a single study ^114^ (5/6 variants), the small size of the dataset (ranging from 28–34 sites) may artificially exacerbate differences between these results. In multiple instances, ASSA has been used to analyze SR proteins, but other programs were used to analyze 5´ and 3´ splice site mutations ^23, 99, 115^. This was surprising, since the donor and acceptor *R _i_* values are generated by default by ASSA and ASSEDA. The advantage of performing both constitutive and regulatory splice site analysis with IT is that all results are reported on the same scale, and the strengths of all interactions, and effects of mutations are directly comparable to one another.

**Table 2a.** Concordance of splice-prediction programs to information theory-based tools for natural and cryptic sites.

	MES ^1^	BDGP ^1^	NG2 ^1^	HSF	SSF ^1, 2^	SSqF ^1^	GS	SV	SP	SS	GenS	ASD	GeneS	GM
**Nat. Donors**	42/48	37/39	24/32	23/28	25/27	15/18	6/11	9/9	5/8	2/2	1/2	1/1	1/1	-
**Nat. Acc.**	21/26	14/19	14/20	12/16	15/18	9/11	3/5	4/5	3/5	-	-	-	-	-
**Cryp. Donors**	16/24	4/8	5/10	16/17	8/8	0/7	2/2	-	-	-	0/1	0/1	-	-
**Cryp. Acc.**	7/13	2/3	3/4	8/11	2/2	2/2	-	-	-	-	-	-	-	0/1
**Neut. Mut.**	31/31	8/8	4/4	26/26	-	-	-	-	-	2/2	-	-	-	-

**Table 2b.** Concordance of splice-prediction programs to information theory-based tools for splicing regulatory proteins.

	ESEfinder ^3, 4^	Rescue-ESE	Ex Skip ^3, 4^	ESEsearch	PESX
**ESEs (all types)**	9/15	3/4	4/14	2/3	1/1
**Neut. Mut.**	4/4	1/1	3/3	-	-

Concordance was assessed from the analysis of variants from 36 publications which used IT-based tools and a secondary predictive method. Each value corresponds to the number of variants that were concordant with IT-based tools versus the total number of variants for each category. ^1^ – includes Vreeswijk *et al.* (2008), which may not have properly reported predicted cryptic sites, as they did not report any cryptic sites predicted by ASSA beyond the default window size (54 nt) from the mutation. ^2^ – predictions made using the SSF-like algorithm in the Alamut splicing prediction module were combined with the SSF category (SSF is no longer supported). ^3^ – Aissat *et al.* (2013) contributes highly to the discordance of these programs, and may be due to improper reporting/analysis. ^4^ – Mutations predicted by alternate program to affect SR protein to which ASSEDA has no model (i.e. 9G8) were not included in statistics.

MES – MaxEntScan; BDGP – Splice Site Prediction by Neural Network, NNSplice; NG2 – NetGene2; HSF – Human Splice Finder; SSF – Splice Site Finder; SSqF – Splicing Sequences Finder; GS - GeneSplicer; SV – SpliceView; SP – Splice Predictor; SS - Shapiro-Senapathy; GenS – GenScan; ASD - ASD-Intron analysis; GeneS – GeneScan; GM – GeneMark; PESX - Putative Exonic Splicing Enhancers/Silencers.

## Other applications of information theory-based splice site analysis

The use of IT to analyze splicing is not limited to sequence variant analysis. The natural and alternative splicing of several genes have been characterized using this method ^107, 200, 240^. Khan *et al.* (2002) scanned all natural sites in the *XPC* gene and found a weak acceptor (-0.1 bits), and with RT-PCR found that this exon (exon 4) was skipped to a greater extent than another (exon 7), which possessed a strong acceptor, illustrating a relationship between the information content of a natural splice site and its level of alternative splicing. IT has also been used in genetic engineering in the design and alteration of binding sites, and has been used in the design of constructs for transgenic animal models ^241– 243^. Thus, IT-based splice site analysis can be adapted for other important molecular genetic applications.

## Guidelines for information theory-based splicing mutation analyses

Our comprehensive review of the use of IT in splicing mutation analysis has led us to propose general recommendations, which we formulate as guidelines. Adoption of these guidelines should ensure the accurate and comprehensive results from IT analyses of VUS and other pathogenic variants that alter mRNA splicing.

### Report gene isoform and genomic coordinates

When analyzing a variant with ASSEDA, the user is prompted to select an mRNA isoform (GenBank or RefSeq accession) from the gene in question. When entering the same variant (in either IVS or c. notation) for different isoforms, either the variant will parse one but not the other representation, or the variant syntax will be processed for both. In the first situation, the user is prompted to verify the position and substitution, which may elicit the realization that the incorrect isoform had been selected. However, in the case where the variant can still be parsed (despite being incorrectly entered for the isoform selected), an incorrect nucleotide may coincidentally have the same sequence, and the user may not necessarily realize that the intended position is not being analyzed. We were unable to reproduce results for several variants, because the mRNA or gene isoform was not reported. This issue could be resolved by comparing the genomic sequence in papers where the context of the mutation was included ^50, 95, 141, 179, 244– 246^. Where flanking sequences were unavailable, the location of the mutation was inferred from either descriptions in the text, the corresponding *R _i_* value of the splice site, or relative coordinate numbering ^144, 247, 248^. Although we attempted to reproduce all the results, this was not always possible if the specified sequence was ambiguous or the source was deprecated (GenBank accession numbers, BAC clones, etc.) ^48, 97, 172, 179, 180, 208, 227, 232, 249, 250^.

We note that ASSA/ASSEDA cannot account for genes with redacted exons, where the exon numbering or sequence in the original mRNA accession has not been corrected. A well-known example is *BRCA1,* for which the constitutive isoform lacks the exon designated as number 4. IVS notation beyond this point in this gene must be reduced by one intron. Alternatively, one of the HGVS-approved methods can be used to input variants, or the variant can be designated with the genomic coordinate (g.) format. Review of ASSA/ASSEDA output (coordinates and/or the sequence walker ^20^) is a prudent approach to confirm that the correct region has been analyzed.

To eliminate ambiguity, we recommend that reported variants be accompanied by the accession number used in its analysis (consistent with HGVS notation ^36^) and the genomic coordinates with the corresponding reference genome build. The table of results from ASSEDA or Shannon pipeline output could also be included as supplementary published material. This will ensure that reported results can be reproduced and compared to other experimental or *in silico* results.

### Report *R _i_* values

The results generated by IT software provide *R _i,initial_*, *R _i,final_*, and Δ *R _i_* for donor and acceptor sites by default, and for all other ribl matrices selected. Reporting these values along with the interpretation improves the clarity of said interpretation. Several publications have not reported *R _i_*, and instead only the interpretation of these values ^125, 138, 146, 212, 227, 251, 252^. This presumes that the analysis was performed correctly, and accurately interpreted. In one instance, our reanalysis differed from the published interpretation ^138^. Other publications provide *R _i_* values, but were incorrectly reported, which resulted in misinterpretations ^48, 122, 253^. Simply reporting Δ *R _i_* itself does not provide sufficient information about the context of the mutation or possible cryptic splice sites, which is necessary to fully appreciate the resultant effect on splicing ^136, 245, 254^. We recommend *R _i_* values be provided for each variant analyzed. We also suggest that the specific donor and acceptor ribl used for variant analysis be indicated, because of the differences obtained using the genome-wide and original PWMs in IT analysis ^30, 33^. The distinction can also be significant, when the *R _i,final_* value of a mutated splice site approaches *R _i,min_*.

### Consider impact of missense and synonymous mutations on mRNA splicing

Missense and synonymous mutations can alter natural splicing, create cryptic sites, and alter crucial ESE and ESS binding sites ^255^. IT tools have been employed to analyze exonic variants that strengthened or create exonic cryptic sites, which were also confirmed experimentally ^25, 39, 41, 43, 98, 105, 116, 124, 130, 149, 151, 178, 256, 257^. Similarly, IT tools can predict potential effects on strengths of SR and hnRNP protein recognition sites ^23, 117^. There is no justification for cataloguing intronic and exonic variants, but only assessing splicing effects for the intronic variants or those within natural splice sites ^119, 132, 175, 186, 208, 210, 214, 215, 248, 258, 259^. We recommend that IT-based analysis should evaluate all variants within a gene for potential splicing mutations.

### Experimentally validate variants

Many studies have reported only coding changes and the results of IT (or other *in silico*) analyses without experimental validation. Our review indicated that IT-based splicing predictions are highly concordant with validation results (87.9%) Nevertheless, the discordant mutations support the need for robust post-prediction validation, since even a single discordant result can lead to misdiagnosis. We do not detect any consistent pattern amongst the discordant predictions to provide guidance as to which IT analyses will be erroneous. Experimental verification will mitigate incorrect interpretations of IT predictions and has been recommended by others ^26^.

### Report the sequence window used in the analysis

ASSA/ASSEDA allows the user to alter size of sequence window range surrounding the mutation. The default window range has been set to maximize the speed of analysis, which is to some degree dictated by the number of matrices and the length of the sequence analyzed. Arbitrary abbreviation of the sequence analysis window can result in the failure to detect activated intronic or exonic cryptic sites, which can in some instances significantly lengthen (eg. 231 and 313 nucleotide extensions, respectively ^166, 171^) or shorten the corresponding natural exon. Therefore, we suggest expanding this window if one wishes to assess the possibility that long range, pre-existing cryptic splice sites may be activated.

We note that unequivocal prediction of cryptic splice site use in large exons (> 1000 nt) can be challenging due to the reliance of these gene regions on splicing enhancers, silencers, and other regulatory elements to prevent ectopic splice site use and ensure fidelity of splicing ^260^. Di Leo *et al.* (2007) determined a variant abolishing the natural acceptor for exon 26 of *APOB* (7572 nt long), which activated a weak cryptic site 1180 nt downstream ^261^. There are several other stronger candidate cryptic splice sites that occur between the natural and cryptic splice site, but there is no evidence that any are used in the individual carrying this mutation.

### Designate genic rearrangements (insertions, deletions, duplications) with genomic coordinates

Complex insertions and deletions in IVS or c. notation may occasionally be parsed to the wrong coordinates within a gene. Indels will parse properly when genomic coordinates are used. If IVS or c. notation is used, it is suggested that users confirm that the expected alteration of the mutation is correct by reviewing the sequence walker display generated by ASSEDA for all insertions, deletions and duplications.

Dataset for mRNA splicing mutations in genetic disease All data from the extensive review of the literature presented in the article are reported as Supplementary tables 1 through 10. The following data are provided: 1) articles referring to information theory as a tool for splice site mutation analysis; 2) complete list of reviewed variants; 3) indels, duplications and multinucleotide variants; 4) deleterious natural site variants; 5) branch point variants; 6–7) Types 1–3 cryptic splice site variants; 9) validated variants; 10) splicing mutation calculator data.

## Data availability

F1000Research: Dataset 1. Dataset for mRNA splicing mutations in genetic disease, 10.5256/f1000research.5654.d38248 ^262^

## Software availability

### Software access

The Splicing Mutation Calculator (SMC) is available at http://splicemc.cytognomix.com.

### Latest source code

https://github.com/F1000Research/splicemc

### Archived source code as at the time of publication

http://dx.doi.org/10.5281/zenodo.12422 ^263^

### License

GNU GPLv3

## Supplementary Material

Supplementary file containing Supplementary Figure 1 and Supplementary Bibliography.

## References

[1] Kan Z Rouchka EC Gish WR: Gene structure prediction and alternative splicing analysis using genomically aligned ESTs. *Genome Res.* 2001;11(5):889–900. 10.1101/gr.155001 11337482 PMC311065

[2] Modrek B Resch A Grasso C: Genome-wide detection of alternative splicing in expressed sequences of human genes. *Nucleic Acids Res.* 2001;29(13):2850–2859. 10.1093/nar/29.13.2850 11433032 PMC55780

[3] Vandenbroucke I Callens T De Paepe A: Complex splicing pattern generates great diversity in human NF1 transcripts. *BMC Genomics.* 2002;3:13. 10.1186/1471-2164-3-13 12057013 PMC115845

[4] Frilander MJ Steitz JA: Initial recognition of U12-dependent introns requires both U11/5’ splice-site and U12/branchpoint interactions. *Genes Dev.* 1999;13(7):851–863. 10.1101/gad.13.7.851 10197985 PMC316595

[5] Will CL Lührmann R: Protein functions in pre-mRNA splicing. *Curr Opin Cell Biol.* 1997;9(3):320–328. 10.1016/S0955-0674(97)80003-8 9159080

[6] Burge CB Tuschi T Sharp PA: The RNA World, 2nd Ed.: The Nature of Modern RNA Suggests a Prebiotic RNA World. *(Cold Spring Harbor Press)* 1999; **37**:525–560 Reference Source

[7] Wu Y Zhang Y Zhang J: Distribution of exonic splicing enhancer elements in human genes. *Genomics.* 2005;86(3):329–336. 10.1016/j.ygeno.2005.05.011 16005179

[8] Graveley BR Hertel KJ Maniatis T: A systematic analysis of the factors that determine the strength of pre-mRNA splicing enhancers. *EMBO J.* 1998;17(22):6747–6756. 10.1093/emboj/17.22.6747 9822617 PMC1171020

[9] Andresen BS Krainer A: When the genetic code is not enough - How sequence variations can affect pre-mRNA splicing and cause (complex) disease. Chapter 15. *Genetics of Complex Human Diseases* (New York, USA: Cold Spring Harbor Laboratory Press),2009 Reference Source

[10] Krawczak M Reiss J Cooper DN: The mutational spectrum of single base-pair substitutions in mRNA splice junctions of human genes: causes and consequences. *Hum Genet.* 1992;90(1–2):41–54. 10.1007/BF00210743 1427786

[11] Ars E Serra E García J: Mutations affecting mRNA splicing are the most common molecular defects in patients with neurofibromatosis type 1. *Hum Mol Genet.* 2000;9(2):237–247. 10.1093/hmg/9.2.237 10607834

[12] Teraoka SN Telatar M Becker-Catania S: Splicing defects in the ataxia-telangiectasia gene, ATM: underlying mutations and consequences. *Am J Hum Genet.* 1999;64(6):1617–1631. 10.1086/302418 10330348 PMC1377904

[13] Shapiro MB Senapathy P: RNA splice junctions of different classes of eukaryotes: sequence statistics and functional implications in gene expression. *Nucleic Acids Res.* 1987;15(17):7155–7174. 10.1093/nar/15.17.7155 3658675 PMC306199

[14] Rogan PK Schneider TD: Using information content and base frequencies to distinguish mutations from genetic polymorphisms in splice junction recognition sites. *Hum Mutat.* 1995;6(1):74–76. 10.1002/humu.1380060114 7550236

[15] Fishel R Lescoe MK Rao MR: The human mutator gene homolog MSH2 and its association with hereditary nonpolyposis colon cancer. *Cell.* 1993;75(5):1027–1038. 10.1016/0092-8674(93)90546-3 8252616

[16] Leach FS Nicolaides NC Papadopoulos N: Mutations of a mutS homolog in hereditary nonpolyposis colorectal cancer. *Cell.* 1993;75(6):1215–1225. 10.1016/0092-8674(93)90330-S 8261515

[17] Schneider TD: Sequence logos, machine/channel capacity, Maxwell’s demon, and molecular computers: a review of the theory of molecular machines. *Nanotechnology.* 1994;5(1):1–18 10.1088/0957-4484/5/1/001

[18] Schneider TD: Information content of individual genetic sequences. *J Theor Biol.* 1997;189(4):427–441. 10.1006/jtbi.1997.0540 9446751

[19] Brunak S Engelbrecht J Knudsen S: Prediction of human mRNA donor and acceptor sites from the DNA sequence. *J Mol Biol.* 1991;220(1):49–65. 10.1016/0022-2836(91)90380-O 2067018

[20] Schneider TD: Sequence walkers: a graphical method to display how binding proteins interact with DNA or RNA sequences. *Nucleic Acids Res.* 1997;25(21):4408–4415. 10.1093/nar/25.21.4408 9336476 PMC147041

[21] Hengen PN Bartram SL Stewart LE: Information analysis of Fis binding sites. *Nucleic Acids Res.* 1997;25(24):4994–5002. 10.1093/nar/25.24.4994 9396807 PMC147151

[22] Rogan PK Faux BM Schneider TD: Information analysis of human splice site mutations. *Hum Mutat.* 1998;12(3):153–171. 10.1002/(SICI)1098-1004(1998)12:3<153::AID-HUMU3>3.0.CO;2-I 9711873

[23] Olsen RKJ Brøner S Sabaratnam R: The ETFDH c.158A>G variation disrupts the balanced interplay of ESE- and ESS-binding proteins thereby causing missplicing and multiple Acyl-CoA dehydrogenation deficiency. *Hum Mutat.* 2014;35(1):86–95. 10.1002/humu.22455 24123825

[24] Homolova K Zavadakova P Doktor TK: The deep intronic c.903+469T>C mutation in the MTRR gene creates an SF2/ASF binding exonic splicing enhancer, which leads to pseudoexon activation and causes the cblE type of homocystinuria. *Hum Mutat.* 2010;31(4):437–444. 10.1002/humu.21206 20120036 PMC3429857

[25] Mucaki EJ Ainsworth P Rogan PK: Comprehensive prediction of mRNA splicing effects of BRCA1 and BRCA2 variants. *Hum Mutat.* 2011;32(7):735–742. 10.1002/humu.21513 21523855

[26] Maddalena A Bale S Das S: Technical standards and guidelines: molecular genetic testing for ultra-rare disorders. *Genet Med.* 2005;7(8):571–583. 10.1097/01.GIM.0000182738.95726.ca 16247296

[27] Shannon CE: A Mathematical Theory of Communication. *Bell Syst Tech J.* 1948;27(3):379–423 10.1002/j.1538-7305.1948.tb01338.x

[28] Schneider TD Stormo GD Gold L: Information content of binding sites on nucleotide sequences. *J Mol Biol.* 1986;188(3):415–431. 10.1016/0022-2836(86)90165-8 3525846

[29] Schneider TD Stephens RM: Sequence logos: a new way to display consensus sequences. *Nucleic Acids Res.* 1990;18(20):6097–6100. 10.1093/nar/18.20.6097 2172928 PMC332411

[30] Rogan PK Svojanovsky S Leeder JS: Information theory-based analysis of CYP2C19, CYP2D6 and CYP3A5 splicing mutations. *Pharmacogenetics.* 2003;13(4):207–218. 10.1097/00008571-200304000-00005 12668917

[31] Schneider TD Stormo GD Haemer JS: A design for computer nucleic-acid-sequence storage, retrieval, and manipulation. *Nucleic Acids Res.* 1982;10(9):3013–3024. 10.1093/nar/10.9.3013 7099972 PMC320671

[32] Schneider TD Stormo GD Yarus MA: Delila system tools. *Nucleic Acids Res.* 1984;12(1 Pt 1):129–140. 10.1093/nar/12.1Part1.129 6694897 PMC320990

[33] Stephens RM Schneider TD: Features of spliceosome evolution and function inferred from an analysis of the information at human splice sites. *J Mol Biol.* 1992;228(4):1124–1136. 10.1016/0022-2836(92)90320-J 1474582

[34] Nalla VK Rogan PK: Automated splicing mutation analysis by information theory. *Hum Mutat.* 2005;25(4):334–342. 10.1002/humu.20151 15776446

[35] Mucaki EJ Shirley BC Rogan PK: Prediction of Mutant mRNA Splice Isoforms by Information Theory-Based Exon Definition. *Hum Mutat.* 2013;34(4):557–565. 10.1002/humu.22277 23348723

[36] Den Dunnen JT Antonarakis SE: Mutation nomenclature extensions and suggestions to describe complex mutations: a discussion. *Hum Mutat.* 2000;15(1):7–12. 10612815 10.1002/(SICI)1098-1004(200001)15:1<7::AID-HUMU4>3.0.CO;2-N

[37] Tribus M: Thermostatics and thermodynamics. (New York: Van Nostrand, 1961). Reference Source

[38] Cover TM Thomas JA: Elements of Information Theory. (John Wiley & Sons).2006 Reference Source

[39] Bonnet-Dupeyron MN Combes P Santander P: PLP1 splicing abnormalities identified in Pelizaeus-Merzbacher disease and SPG2 fibroblasts are associated with different types of mutations. *Hum Mutat.* 2008;29(8):1028–1036. 10.1002/humu.20758 18470932

[40] Rogan PK Zou GY: Best practices for evaluating mutation prediction methods. *Hum Mutat.* 2013;34(11):1581–1582. 10.1002/humu.22401 23955774

[41] Shirley BC Mucaki EJ Whitehead T: Interpretation, stratification and evidence for sequence variants affecting mRNA splicing in complete human genome sequences. *Genomics Proteomics Bioinformatics.* 2013;11(2):77–85. 10.1016/j.gpb.2013.01.008 23499923 PMC4357664

[42] Benaglio P San Jose PF Avila-Fernandez A: Mutational screening of splicing factor genes in cases with autosomal dominant retinitis pigmentosa. *Mol Vis.* 2014;20:843–851. 24959063 PMC4063357

[43] Viner C Dorman SN Shirley BC: Validation of predicted mRNA splicing mutations using high-throughput transcriptome data. *F1000Res.* 2014;3:8. 10.12688/f1000research.3-8.v2 24741438 PMC3983938

[44] Dorman S Viner C Rogan P: Splicing Mutation Analysis Reveals Previously Unrecognized Pathways in Lymph Node-Invasive Breast Cancer. In Press. *Sci Rep.* 2014. 10.1038/srep07063 PMC4231324 25394353

[45] Green MR: Pre-mRNA splicing. *Annu Rev Genet.* 1986;20:671–708. 10.1146/annurev.ge.20.120186.003323 2880558

[46] Maniatis T Reed R: The role of small nuclear ribonucleoprotein particles in pre-mRNA splicing. *Nature.* 1987;325(6106):673–678. 10.1038/325673a0 2950324

[47] Khan SG Metin A Gozukara E: Two essential splice lariat branchpoint sequences in one intron in a xeroderma pigmentosum DNA repair gene: mutations result in reduced XPC mRNA levels that correlate with cancer risk. *Hum Mol Genet.* 2004;13(3):343–352. 10.1093/hmg/ddh026 14662655

[48] Fei J: Splice Site Mutation-Induced Alteration of Selective Regional Activity Correlates with the Role of a Gene in Cardiomyopathy. *J Clin Exp Cardiol.* 2013;1.

[49] Robberson BL Cote GJ Berget SM: Exon definition may facilitate splice site selection in RNAs with multiple exons. *Mol Cell Biol.* 1990;10(1):84–94. 10.1128/MCB.10.1.84 2136768 PMC360715

[50] Smaoui N Beltaief O BenHamed S: A homozygous splice mutation in the HSF4 gene is associated with an autosomal recessive congenital cataract. *Invest Ophthalmol Vis Sci.* 2004;45(8):2716–2721. 10.1167/iovs.03-1370 15277496

[51] Sharma N Sosnay PR Ramalho AS: Experimental assessment of splicing variants using expression minigenes and comparison with *in silico* predictions. *Hum Mutat.* 2014;35(10):1249–1259. 10.1002/humu.22624 25066652 PMC4425124

[52] Riveira-Munoz E Devuyst O Belge H: Evaluating PVALB as a candidate gene for SLC12A3-negative cases of Gitelman’s syndrome. *Nephrol Dial Transplant.* 2008;23(10):3120–3125. 10.1093/ndt/gfn229 18469313

[53] Ozaltin F Ibsirlioglu T Taskiran EZ: Disruption of PTPRO causes childhood-onset nephrotic syndrome. *Am J Hum Genet.* 2011;89(1):139–147. 10.1016/j.ajhg.2011.05.026 21722858 PMC3135805

[54] Di Leo E Magnolo L Pinotti E: Functional analysis of two novel splice site mutations of APOB gene in familial hypobetalipoproteinemia. *Mol Genet Metab.* 2009;96(2):66–72. 10.1016/j.ymgme.2008.10.016 19084451

[55] Behzadnia N Golas MM Hartmuth K: Composition and three-dimensional EM structure of double affinity-purified, human prespliceosomal A complexes. *EMBO J.* 2007;26(6):1737–1748. 10.1038/sj.emboj.7601631 17332742 PMC1829389

[56] Staley JP Guthrie C: Mechanical devices of the spliceosome: motors, clocks, springs, and things. *Cell.* 1998;92(3):315–326. 10.1016/S0092-8674(00)80925-3 9476892

[57] Krawczak M Thomas NS Hundrieser B: Single base-pair substitutions in exon-intron junctions of human genes: nature, distribution, and consequences for mRNA splicing. *Hum Mutat.* 2007;28(2):150–158. 10.1002/humu.20400 17001642

[58] Mort M Sterne-Weiler T Li B: MutPred Splice: machine learning-based prediction of exonic variants that disrupt splicing. *Genome Biol.* 2014;15(1):R19. 10.1186/gb-2014-15-1-r19 24451234 PMC4054890

[59] Sun H Chasin LA: Multiple splicing defects in an intronic false exon. *Mol Cell Biol.* 2000;20(17):6414–6425. 10.1128/MCB.20.17.6414-6425.2000 10938119 PMC86117

[60] Treisman R Orkin SH Maniatis T: Specific transcription and RNA splicing defects in five cloned beta-thalassaemia genes. *Nature.* 1983;302(5909):591–596. 10.1038/302591a0 6188062

[61] ElSharawy A Hundrieser B Brosch M: Systematic evaluation of the effect of common SNPs on pre-mRNA splicing. *Hum Mutat.* 2009;30(4):625–632. 10.1002/humu.20906 19191320

[62] Buratti E Baralle M Baralle FE: Defective splicing, disease and therapy: searching for master checkpoints in exon definition. *Nucleic Acids Res.* 2006;34(12):3494–3510. 10.1093/nar/gkl498 16855287 PMC1524908

[63] Kapustin Y Chan E Sarkar R: Cryptic splice sites and split genes. *Nucleic Acids Res.* 2011;39(14):5837–5844. 10.1093/nar/gkr203 21470962 PMC3152350

[64] Zhang MQ: Computational prediction of eukaryotic protein-coding genes. *Nat Rev Genet.* 2002;3(9):698–709. 10.1038/nrg890 12209144

[65] Fu XD: The superfamily of arginine/serine-rich splicing factors. *RNA.* 1995;1(7):663–680. 7585252 PMC1369309

[66] Graveley BR: Sorting out the complexity of SR protein functions. *RNA.* 2000;6(9):1197–1211. 10.1017/S1355838200000960 10999598 PMC1369994

[67] Sharma S Kohlstaedt LA Damianov A: Polypyrimidine tract binding protein controls the transition from exon definition to an intron defined spliceosome. *Nat Struct Mol Biol.* 2008;15(2):183–191. 10.1038/nsmb.1375 18193060 PMC2546704

[68] Zheng ZM Huynen M Baker CC: A pyrimidine-rich exonic splicing suppressor binds multiple RNA splicing factors and inhibits spliceosome assembly. *Proc Natl Acad Sci U S A.* 1998;95(24):14088–14093. 10.1073/pnas.95.24.14088 9826658 PMC24331

[69] House AE Lynch KW: An exonic splicing silencer represses spliceosome assembly after ATP-dependent exon recognition. *Nat Struct Mol Biol.* 2006;13(10):937–944. 10.1038/nsmb1149 16998487

[70] Shen M Mattox W: Activation and repression functions of an SR splicing regulator depend on exonic versus intronic-binding position. *Nucleic Acids Res.* 2012;40(1):428–437. 10.1093/nar/gkr713 21914724 PMC3245930

[71] Erkelenz S Mueller WF Evans MS: Position-dependent splicing activation and repression by SR and hnRNP proteins rely on common mechanisms. *RNA.* 2013;19(1):96–102. 10.1261/rna.037044.112 23175589 PMC3527730

[72] Zatkova A Messiaen L Vandenbroucke I: Disruption of exonic splicing enhancer elements is the principal cause of exon skipping associated with seven nonsense or missense alleles of NF1. *Hum Mutat.* 2004;24(6):491–501. 10.1002/humu.20103 15523642

[73] Gonçalves V Theisen P Antunes O: A missense mutation in the APC tumor suppressor gene disrupts an ASF/SF2 splicing enhancer motif and causes pathogenic skipping of exon 14. *Mutat Res.* 2009;662(1–2):33–36. 10.1016/j.mrfmmm.2008.12.001 19111562

[74] Miyajima H Miyaso H Okumura M: Identification of a cis-acting element for the regulation of SMN exon 7 splicing. *J Biol Chem.* 2002;277(26):23271–23277. 10.1074/jbc.M200851200 11956196

[75] Burgess R MacLaren RE Davidson AE: ADVIRC is caused by distinct mutations in BEST1 that alter pre-mRNA splicing. *J Med Genet.* 2009;46(9):620–625. 10.1136/jmg.2008.059881 18611979

[76] Gabut M Miné M Marsac C: The SR protein SC35 is responsible for aberrant splicing of the E1alpha pyruvate dehydrogenase mRNA in a case of mental retardation with lactic acidosis. *Mol Cell Biol.* 2005;25(8):3286–3294. 10.1128/MCB.25.8.3286-3294.2005 15798212 PMC1069624

[77] Goren A Kim E Amit M: Overlapping splicing regulatory motifs--combinatorial effects on splicing. *Nucleic Acids Res.* 2010;38(10):3318–3327. 10.1093/nar/gkq005 20110253 PMC2879502

[78] Zahler AM Damgaard CK Kjems J: SC35 and heterogeneous nuclear ribonucleoprotein A/B proteins bind to a juxtaposed exonic splicing enhancer/exonic splicing silencer element to regulate HIV-1 tat exon 2 splicing. *J Biol Chem.* 2004;279(11):10077–10084. 10.1074/jbc.M312743200 14703516

[79] Chou MY Rooke N Turck CW: hnRNP H is a component of a splicing enhancer complex that activates a c-src alternative exon in neuronal cells. *Mol Cell Biol.* 1999;19(1):69–77. 9858532 10.1128/mcb.19.1.69 PMC83866

[80] Xu J Lu Z Xu M: A heroin addiction severity-associated intronic single nucleotide polymorphism modulates alternative pre-mRNA splicing of the μ opioid receptor gene OPRM1 via hnRNPH interactions. *J Neurosci.* 2014;34(33):11048–11066. 10.1523/JNEUROSCI.3986-13.2014 25122903 PMC4131016

[81] Fu XD Ares M Jr: Context-dependent control of alternative splicing by RNA-binding proteins. *Nat Rev Genet.* 2014;15(10):689–701. 10.1038/nrg3778 25112293 PMC4440546

[82] Mercado PA Ayala YM Romano M: Depletion of TDP 43 overrides the need for exonic and intronic splicing enhancers in the human apoA-II gene. *Nucleic Acids Res.* 2005;33(18):6000–6010. 10.1093/nar/gki897 16254078 PMC1270946

[83] Huelga SC Vu AQ Arnold JD: Integrative genome-wide analysis reveals cooperative regulation of alternative splicing by hnRNP proteins. *Cell Rep.* 2012;1(2):167–178. 10.1016/j.celrep.2012.02.001 22574288 PMC3345519

[84] Tavanez JP Madl T Kooshapur H: hnRNP A1 proofreads 3´ splice site recognition by U2AF. *Mol Cell.* 2012;45(3):314–329. 10.1016/j.molcel.2011.11.033 22325350

[85] Caputi M Freund M Kammler S: A bidirectional SF2/ASF- and SRp40-dependent splicing enhancer regulates human immunodeficiency virus type 1 rev, env, vpu, and nef gene expression. *J Virol.* 2004;78(12):6517–6526. 10.1128/JVI.78.12.6517-6526.2004 15163745 PMC416506

[86] Expert-Bezançon A Sureau A Durosay P: hnRNP A1 and the SR proteins ASF/SF2 and SC35 have antagonistic functions in splicing of beta-tropomyosin exon 6B. *J Biol Chem.* 2004;279(37):38249–38259. 10.1074/jbc.M405377200 15208309

[87] Liu HX Chew SL Cartegni L: Exonic splicing enhancer motif recognized by human SC35 under splicing conditions. *Mol Cell Biol.* 2000;20(3):1063–1071. 10.1128/MCB.20.3.1063-1071.2000 10629063 PMC85223

[88] Pandit S Zhou Y Shiue L: Genome-wide analysis reveals SR protein cooperation and competition in regulated splicing. *Mol Cell.* 2013;50(2):223–235. 10.1016/j.molcel.2013.03.001 23562324 PMC3640356

[89] Han J Ding JH Byeon CW: SR proteins induce alternative exon skipping through their activities on the flanking constitutive exons. *Mol Cell Biol.* 2011;31(4):793–802. 10.1128/MCB.01117-10 21135118 PMC3028638

[90] Shultz JC Goehe RW Murudkar CS: SRSF1 regulates the alternative splicing of caspase 9 via a novel intronic splicing enhancer affecting the chemotherapeutic sensitivity of non-small cell lung cancer cells. *Mol Cancer Res.* 2011;9(7):889–900. 10.1158/1541-7786.MCR-11-0061 21622622 PMC3140550

[91] Paradis C Cloutier P Shkreta L: hnRNP I/PTB can antagonize the splicing repressor activity of SRp30c. *RNA.* 2007;13(8):1287–1300. 10.1261/rna.403607 17548433 PMC1924885

[92] Mukherjee N Corcoran DL Nusbaum JD: Integrative regulatory mapping indicates that the RNA-binding protein HuR couples pre-mRNA processing and mRNA stability. *Mol Cell.* 2011;43(3):327–339. 10.1016/j.molcel.2011.06.007 21723170 PMC3220597

[93] Uren PJ Burns SC Ruan J: Genomic analyses of the RNA-binding protein Hu antigen R (HuR) identify a complex network of target genes and novel characteristics of its binding sites. *J Biol Chem.* 2011;286(43):37063–37066. 10.1074/jbc.C111.266882 21890634 PMC3199453

[94] Kölsch H Lütjohann D Jessen F: CYP46A1 variants influence Alzheimer’s disease risk and brain cholesterol metabolism. *Eur Psychiatry.* 2009;24(3):183–190. 10.1016/j.eurpsy.2008.12.005 19286353

[95] Khan SG Levy HL Legerski R: Xeroderma pigmentosum group C splice mutation associated with autism and hypoglycinemia. *J Invest Dermatol.* 1998;111(5):791–796. 10.1046/j.1523-1747.1998.00391.x 9804340

[96] Maruszak A Safranow K Gustaw K: PIN1 gene variants in Alzheimer’s disease. *BMC Med Genet.* 2009;10:115. 10.1186/1471-2350-10-115 19909517 PMC2781804

[97] Caux-Moncoutier V Pagès-Berhouet S Michaux D: Impact of BRCA1 and BRCA2 variants on splicing: clues from an allelic imbalance study. *Eur J Hum Genet.* 2009;17(11):1471–1480. 10.1038/ejhg.2009.89 19471317 PMC2986693

[98] Vockley J Rogan PK Anderson BD: Exon skipping in IVD RNA processing in isovaleric acidemia caused by point mutations in the coding region of the IVD gene. *Am J Hum Genet.* 2000;66(2):356–367. 10.1086/302751 10677295 PMC1288088

[99] Vemula SR Xiao J Zhao Y: A rare sequence variant in intron 1 of THAP1 is associated with primary dystonia. *Mol Genet Genomic Med.* 2014;2(3):261–272. 10.1002/mgg3.67 24936516 PMC4049367

[100] Astuto LM Kelley PM Askew JW: Searching for evidence of DFNB2. *Am J Med Genet.* 2002;109(4):291–297. 10.1002/ajmg.10384 11992483

[101] López-Jiménez E de Campos JM Kusak EM: *SDHC* mutation in an elderly patient without familial antecedents. *Clin Endocrinol (Oxf).* 2008;69(6):906–910. 10.1111/j.1365-2265.2008.03368.x 18681855

[102] Baturina OA Lukjanova TV Tupikin AE: PAH And QDPR Deficiency Associated Mutations In The Novosirirsk Rregion Of The Russian Federation: Correlation Of Mutation Type With Sisease Manifestation And Severity. *J Med Biochem.* 2014;33(4):7–14 10.2478/jomb-2014-0019

[103] Dash DP George S O’Prey D: Mutational screening of *VSX1* in keratoconus patients from the European population. *Eye (Lond).* 2010;24(6):1085–1092. 10.1038/eye.2009.217 19763142

[104] Ellis JR Jr Heinrich B Mautner VF: Effects of splicing mutations on *NF2*-transcripts: transcript analysis and information theoretic predictions. *Genes Chromosomes Cancer.* 2011;50(8):571–584. 10.1002/gcc.20876 21563229 PMC3142993

[105] Wessagowit V Kim SC Woong Oh S: Genotype-phenotype correlation in recessive dystrophic epidermolysis bullosa: when missense doesn't make sense. *J Invest Dermatol.* 2005;124(4):863–866. 10.1111/j.0022-202X.2005.23650.x 15816848

[106] Chang YF Imam JS Wilkinson MF: The nonsense-mediated decay RNA surveillance pathway. *Annu Rev Biochem.* 2007;76:51–74. 10.1146/annurev.biochem.76.050106.093909 17352659

[107] Khan SG Muniz-Medina V Shahlavi T: The human *XPC* DNA repair gene: arrangement, splice site information content and influence of a single nucleotide polymorphism in a splice acceptor site on alternative splicing and function. *Nucleic Acids Res.* 2002;30(16):3624–3631. 10.1093/nar/gkf469 12177305 PMC134237

[108] Goldin E Stahl S Cooney AM: Transfer of a mitochondrial DNA fragment to *MCOLN1* causes an inherited case of mucolipidosis IV. *Hum Mutat.* 2004;24(6):460–465. 10.1002/humu.20094 15523648

[109] Bloethner S Mould A Stark M: Identification of *ARHGEF17*, *DENND2D*, *FGFR3*, and *RB1* mutations in melanoma by inhibition of nonsense-mediated mRNA decay. *Genes Chromosomes Cancer.* 2008;47(12):1076–1085. 10.1002/gcc.20598 18677770

[110] Denson J Xi Z Wu Y: Screening for inter-individual splicing differences in human GSTM4 and the discovery of a single nucleotide substitution related to the tandem skipping of two exons. *Gene.* 2006;379:148–155. 10.1016/j.gene.2006.05.012 16854533

[111] Ben-Salem S Begum MA Ali BR: A Novel Aberrant Splice Site Mutation in *RAB23* Leads to an Eight Nucleotide Deletion in the mRNA and Is Responsible for Carpenter Syndrome in a Consanguineous Emirati Family. *Mol Syndromol.* 2013;3(6):255–261. 10.1159/000345653 23599695 PMC3569107

[112] Aggarwal S Jinda W Limwongse C: Run-on mutation in the PAX6 gene and chorioretinal degeneration in autosomal dominant aniridia. *Mol Vis.* 2011;17:1305–1309. 21633710 PMC3103739

[113] Di Giacomo D Gaildrat P Abuli A: Functional analysis of a large set of *BRCA2* exon 7 variants highlights the predictive value of hexamer scores in detecting alterations of exonic splicing regulatory elements. *Hum Mutat.* 2013;34(11):1547–1557. 10.1002/humu.22428 23983145

[114] Aissat A de Becdelièvre A Golmard L: Combined computational-experimental analyses of *CFTR* exon strength uncover predictability of exon-skipping level. *Hum Mutat.* 2013;34(6):873–881. 10.1002/humu.22300 23420618

[115] Anczuków O Buisson M Salles MJ: Unclassified variants identified in *BRCA1* exon 11: Consequences on splicing. *Genes Chromosomes Cancer.* 2008;47(5):418–426. 10.1002/gcc.20546 18273839

[116] Colombo M De Vecchi G Caleca L: Comparative *in vitro* and *in silico* analyses of variants in splicing regions of *BRCA1* and *BRCA2* genes and characterization of novel pathogenic mutations. *PloS One.* 2013;8(2):e57173. 10.1371/journal.pone.0057173 23451180 PMC3579815

[117] Lacroix M Lacaze-Buzy L Furio L: Clinical expression and new *SPINK5* splicing defects in Netherton syndrome: unmasking a frequent founder synonymous mutation and unconventional intronic mutations. *J Invest Dermatol.* 2012;132(3 Pt 1):575–582. 10.1038/jid.2011.366 22089833

[118] Lamba V Lamba J Yasuda K: Hepatic CYP2B6 expression: gender and ethnic differences and relationship to *CYP2B6* genotype and CAR (constitutive androstane receptor) expression. *J Pharmacol Exp Ther.* 2003;307(3):906–922. 10.1124/jpet.103.054866 14551287

[119] Lee YW Lee DH Vockley J: Different spectrum of mutations of isovaleryl-CoA dehydrogenase ( *IVD*) gene in Korean patients with isovaleric acidemia. *Mol Genet Metab.* 2007;92(1–2):71–77. 10.1016/j.ymgme.2007.05.003 17576084 PMC4136440

[120] Le Guédard-Méreuze S Vaché C Molinari N: Sequence contexts that determine the pathogenicity of base substitutions at position +3 of donor splice-sites. *Hum Mutat.* 2009;30(9):1329–1339. 10.1002/humu.21070 19606495

[121] Tournier I Vezain M Martins A: A large fraction of unclassified variants of the mismatch repair genes *MLH1* and *MSH2* is associated with splicing defects. *Hum Mutat.* 2008;29(12):1412–1424. 10.1002/humu.20796 18561205

[122] Laššuthová P Zaliová M Inoue K: Three New *PLP1* Splicing Mutations Demonstrate Pathogenic and Phenotypic Diversity of Pelizaeus-Merzbacher Disease. *J Child Neurol.* 2013;29(7):924–931. 10.1177/0883073813492387 23771846

[123] Hefferon TW Broackes-Carter FC Harris A: Atypical 5’ splice sites cause CFTR exon 9 to be vulnerable to skipping. *Am J Hum Genet.* 2002;71(2):294–303. 10.1086/341664 12068373 PMC379162

[124] O’Neill JP Rogan PK Cariello N: Mutations that alter RNA splicing of the human *HPRT* gene: a review of the spectrum. *Mutat. Res.* 1998;411(3):179–214. 10.1016/S1383-5742(98)00013-1 9804951

[125] Nasim MT Ogo T Ahmed M: Molecular genetic characterization of SMAD signaling molecules in pulmonary arterial hypertension. *Hum Mutat.* 2011;32(12):1385–1389. 10.1002/humu.21605 21898662

[126] Bocchi L Pisciotta L Fasano T: Multiple abnormally spliced ABCA1 mRNAs caused by a novel splice site mutation of ABCA1 gene in a patient with Tangier disease. *Clin Chim Acta.* 2010;411(7–8):524–530. 10.1016/j.cca.2010.01.008 20093111

[127] Von Kodolitsch Y Berger J Rogan PK: Predicting severity of haemophilia A and B splicing mutations by information analysis. *Haemophilia.* 2006;12(3):258–262. 10.1111/j.1365-2516.2006.01216.x 16643211

[128] Hageman GS Anderson DH Johnson LV: A common haplotype in the complement regulatory gene factor H ( *HF1/CFH*) predisposes individuals to age-related macular degeneration. *Proc Natl Acad Sci U S A.* 2005;102(20):7227–7232. 10.1073/pnas.0501536102 15870199 PMC1088171

[129] Ben Selma Z Yilmaz S Schischmanoff PO: A novel S115G mutation of CGI-58 in a Turkish patient with Dorfman-Chanarin syndrome. *J Invest Dermatol.* 2007;127(9):2273–2276. 10.1038/sj.jid.5700860 17495960

[130] Roux-Buisson N Rendu J Denjoy I: Functional analysis reveals splicing mutations of the *CASQ2* gene in patients with CPVT: implication for genetic counselling and clinical management. *Hum Mutat.* 2011;32(9):995–999. 10.1002/humu.21537 21618644

[131] Qin S Shen L Zhang A: Systematic polymorphism analysis of the *CYP2D6* gene in four different geographical Han populations in mainland China. *Genomics.* 2008;92(3):152–158. 10.1016/j.ygeno.2008.05.004 18632250

[132] Gaweda-Walerych K Safranow K Maruszak A: Mitochondrial transcription factor A variants and the risk of Parkinson’s disease. *Neurosci Lett.* 2010;469(1):24–29. 10.1016/j.neulet.2009.11.037 19925850

[133] Fornage M Lee CR Doris PA: The soluble epoxide hydrolase gene harbors sequence variation associated with susceptibility to and protection from incident ischemic stroke. *Hum Mol Genet.* 2005;14(19):2829–2837. 10.1093/hmg/ddi315 16115816 PMC1343524

[134] Allikmets R Wasserman WW Hutchinson A: Organization of the *ABCR* gene: analysis of promoter and splice junction sequences. *Gene.* 1998;215(1):111–122. 10.1016/S0378-1119(98)00269-8 9666097

[135] Simpson MA Hsu R Keir LS: Mutations in FAM20C are associated with lethal osteosclerotic bone dysplasia (Raine syndrome), highlighting a crucial molecule in bone development. *Am J Hum Genet.* 2007;81(5):906–912. 17924334 10.1086/522240 PMC2265657

[136] Henneman P Schaap FG Rensen PC: Estrogen induced hypertriglyceridemia in an apolipoprotein AV deficient patient. *J Intern Med.* 2008;263(1):107–108. 10.1111/j.1365-2796.2007.01889.x 18088255

[137] Fong K Rama Devi AR Lai-Cheong JE: Infantile systemic hyalinosis associated with a putative splice-site mutation in the *ANTXR2* gene. *Clin Exp Dermatol.* 2012;37(6):635–638. 10.1111/j.1365-2230.2011.04287.x 22300424

[138] Douglas DA Zhong H Ro JY: Novel mutations of epidermal growth factor receptor in localized prostate cancer. *Front Biosci.* 2006;11:2518–2525. 16720329 10.2741/1986

[139] Gaedigk A Baker DW Totah RA: Variability of CYP2J2 expression in human fetal tissues. *J Pharmacol Exp Ther.* 2006;319(2):523–532. 10.1124/jpet.106.109215 16868033 PMC1876721

[140] Sabet A Li J Ghandour K: Skin biopsies demonstrate MPZ splicing abnormalities in Charcot-Marie-Tooth neuropathy 1B. *Neurology.* 2006;67(7):1141–1146. 17030746 10.1212/01.wnl.0000238499.37764.b1

[141] Concolino P Vendittelli F Mello E: Functional analysis of two rare *CYP21A2* mutations detected in Italian patients with a mildest form of congenital adrenal hyperplasia. *Clin Endocrinol (Oxf).* 2009;71(4):470–476. 10.1111/j.1365-2265.2008.03517.x 19170707

[142] Marras E Willems P Vandersickel V: Discrepancies between *in silico* and *in vitro* data in the functional analysis of a breast cancer-associated polymorphism in the XRCC6/Ku70 gene. *Mol Med Rep.* 2008;1(6):805–812. 10.3892/mmr_00000032 21479489

[143] Li A Jiao X Munier FL: Bietti crystalline corneoretinal dystrophy is caused by mutations in the novel gene *CYP4V2*. *Am J Hum Genet.* 2004;74(5):817–826. 15042513 10.1086/383228 PMC1181977

[144] Borroni B Archetti S Alberici A: Progranulin genetic variations in frontotemporal lobar degeneration: evidence for low mutation frequency in an Italian clinical series. *Neurogenetics.* 2008;9(3):197–205. 10.1007/s10048-008-0127-3 18392865

[145] Kölsch H Lütjohann D Jessen F: RXRA gene variations influence Alzheimer’s disease risk and cholesterol metabolism. *J Cell Mol Med.* 2009;13(3):589–598. 10.1111/j.1582-4934.2009.00383.x 19374686 PMC3822518

[146] Jeon GW Kwon MJ Lee SJ: Clinical and genetic analysis of a Korean patient with X-linked chondrodysplasia punctata: identification of a novel splicing mutation in the ARSE gene. *Ann Clin Lab Sci.* 2013;43(1):70–75. 23462608

[147] Vreeswijk MP Kraan JN van der Klift HM: Intronic variants in *BRCA1* and *BRCA2* that affect RNA splicing can be reliably selected by splice-site prediction programs. *Hum Mutat.* 2009;30(1):107–114. 10.1002/humu.20811 18693280

[148] Kölsch H Jessen F Wiltfang J: Association of SORL1 gene variants with Alzheimer’s disease. *Brain Res.* 2009;1264:1–6. 10.1016/j.brainres.2009.01.044 19368828

[149] Oh SW Lee JS Kim MY: *COL7A1* mutational analysis in Korean patients with dystrophic epidermolysis bullosa. *Br J Dermatol.* 2007;157(6):1260–1264. 10.1111/j.1365-2133.2007.08191.x 17916216

[150] Sanggaard KM Rendtorff ND Kjaer KW: Branchio-oto-renal syndrome: detection of *EYA1* and *SIX1* mutations in five out of six Danish families by combining linkage, MLPA and sequencing analyses. *Eur J Hum Genet.* 2007;15(11):1121–1131. 10.1038/sj.ejhg.5201900 17637804

[151] Wessagowit V Nalla VK Rogan PK: Normal and abnormal mechanisms of gene splicing and relevance to inherited skin diseases. *J Dermatol Sci.* 2005;40(2):73–84. 10.1016/j.jdermsci.2005.05.006 16054339 PMC1351063

[152] Slavotinek AM Baranzini SE Schanze D: Manitoba-oculo-tricho-anal (MOTA) syndrome is caused by mutations in FREM1. *J Med Genet.* 2011;48(6):375–382. 10.1136/jmg.2011.089631 21507892 PMC4294942

[153] Moriwaki K Noda K Furukawa Y: Deficiency of GMDS leads to escape from NK cell-mediated tumor surveillance through modulation of TRAIL signaling. *Gastroenterology.* 2009;137(1):188–198, 198.e1–2. 10.1053/j.gastro.2009.04.002 19361506

[154] Bröer S Bailey CG Kowalczuk S: Iminoglycinuria and hyperglycinuria are discrete human phenotypes resulting from complex mutations in proline and glycine transporters. *J Clin Invest.* 2008;118(12):3881–3892. 10.1172/JCI36625 19033659 PMC2579706

[155] Kwon MJ Baek W Ki CS: Screening of the SOD1, FUS, TARDBP, ANG, and OPTN mutations in Korean patients with familial and sporadic ALS. *Neurobiol Aging.* 2012;33(5):1017.e17–23. 10.1016/j.neurobiolaging.2011.12.003 22244934

[156] Clark GR Crowe P Muszynska D: Development of a diagnostic genetic test for simplex and autosomal recessive retinitis pigmentosa. *Ophthalmology.* 2010;117(11):2169–2177.e3. 10.1016/j.ophtha.2010.02.029 20591486

[157] Bertolini S Pisciotta L Rabacchi C: Spectrum of mutations and phenotypic expression in patients with autosomal dominant hypercholesterolemia identified in Italy. *Atherosclerosis.* 2013;227(2):342–348. 10.1016/j.atherosclerosis.2013.01.007 23375686

[158] Catucci I Peterlongo P Ciceri S: PALB2 sequencing in Italian familial breast cancer cases reveals a high-risk mutation recurrent in the province of Bergamo. *Genet Med* 2014;16(9):688–694. 10.1038/gim.2014.13 24556926

[159] Faustino NA Cooper TA: Pre-mRNA splicing and human disease. *Genes Dev.* 2003;17(4):419–437. 10.1101/gad.1048803 12600935

[160] Wang P Guo X Jia X: Novel mutations of the PAX6 gene identified in Chinese patients with aniridia. *Mol Vis.* 2006;12:644–648. 16785853

[161] Hamada T Fukuda S Sakaguchi S: Molecular and clinical characterization in Japanese and Korean patients with Hailey-Hailey disease: six new mutations in the ATP2C1 gene. *J Dermatol Sci.* 2008;51(1):31–36. 10.1016/j.jdermsci.2008.02.003 18372165

[162] Baturina OA Tupikin AE Lukjanova TV: PAH and QDPR Deficiency Associated Mutations in the Novosibirsk Region of the Russian Federation: Correlation of Mutation Type with Disease Manifestation and Severity. *J Med Biochem.* 2014;33(4):333–340 10.2478/jomb-2014-0019

[163] Yu H Patel SB: Recent insights into the Smith-Lemli-Opitz syndrome. *Clin Genet.* 2005;68(5):383–391. 10.1111/j.1399-0004.2005.00515.x 16207203 PMC1350989

[164] Russcher H Smit P van Rossum EF: Strategies for the characterization of disorders in cortisol sensitivity. *J Clin Endocrinol Metab.* 2006;91(2):694–701. 10.1210/jc.2005-2212 16317053

[165] Leclerc D Wu Q Ellis JR: Is the SLC7A10 gene on chromosome 19 a candidate locus for cystinuria? *Mol Genet Metab.* 2001;73(4):333–339. 10.1006/mgme.2001.3209 11509015

[166] Leclerc D Boutros M Suh D: SLC7A9 mutations in all three cystinuria subtypes. *Kidney Int.* 2002;62(5):1550–1559. 10.1046/j.1523-1755.2002.00602.x 12371955

[167] Marchal A Goffinetb L Charlesworth A: Un cas particulier d’épidermolyse bulleuse dystrophique. *Ann Dermatol Venereol.* 2011;138(12):A168–A169 10.1016/j.annder.2011.10.114

[168] von Kodolitsch Y Pyeritz RE Rogan PK: Splice-Site mutations in atherosclerosis candidate genes: relating individual information to phenotype. *Circulation.* 1999;100(7):693–699. 10.1161/01.CIR.100.7.693 10449689

[169] Oh KS Khan SG Jaspers NG: Phenotypic heterogeneity in the XPB DNA helicase gene (ERCC3): xeroderma pigmentosum without and with Cockayne syndrome. *Hum Mutat.* 2006;27(11):1092–1103. 10.1002/humu.20392 16947863

[170] Lim BC Ki CS Kim JW: Fukutin mutations in congenital muscular dystrophies with defective glycosylation of dystroglycan in Korea. *Neuromuscul Disord NMD.* 2010;20(8):524–530. 10.1016/j.nmd.2010.06.005 20620061

[171] Marco EJ Abidi FE Bristow J: ARHGEF9 disruption in a female patient is associated with X linked mental retardation and sensory hyperarousal. *J Med Genet.* 2008;45(2):100–105. 17893116 10.1136/jmg.2007.052324

[172] Gemignani F Moreno V Landi S: A TP53 polymorphism is associated with increased risk of colorectal cancer and with reduced levels of TP53 mRNA. *Oncogene.* 2003;23(10):1954–1956. 10.1038/sj.onc.1207305 14647431

[173] Luquin N Yu B Saunderson RB: Genetic variants in the promoter of TARDBP in sporadic amyotrophic lateral sclerosis. *Neuromuscul Disord.* 2009;19(10):696–700. 10.1016/j.nmd.2009.07.005 19695877

[174] Magnolo L Najah M Fancello T: Novel mutations in SAR1B and MTTP genes in Tunisian children with chylomicron retention disease and abetalipoproteinemia. *Gene.* 2013;512(1):28–34. 10.1016/j.gene.2012.09.117 23043934

[175] Marr N Bichet DG Hoefs S: Cell-biologic and functional analyses of five new Aquaporin-2 missense mutations that cause recessive nephrogenic diabetes insipidus. *J Am Soc Nephrol.* 2002;13(9):2267–2277. 10.1097/01.ASN.0000027355.41663.14 12191971

[176] Naiya T Misra AK Biswas A: Occurrence of GCH1 gene mutations in a group of Indian dystonia patients. *J Neural Transm.* 2012;119(11):1343–1350. 10.1007/s00702-012-0777-z 22373569

[177] Fasano T Bocchi L Pisciotta L: Denaturing high-performance liquid chromatography in the detection of ABCA1 gene mutations in familial HDL deficiency. *J Lipid Res.* 2005;46(4):817–822. 10.1194/jlr.D400038-JLR200 15722566

[178] Tosetto E Ghiggeri GM Emma F: Phenotypic and genetic heterogeneity in Dent’s disease--the results of an Italian collaborative study. *Nephrol Dial Transplant.* 2006;21(9):2452–2463. 10.1093/ndt/gfl274 16822791

[179] Tosetto E Ceol M Mezzabotta F: Novel mutations of the CLCN5 gene including a complex allele and A 5’ UTR mutation in Dent disease 1. *Clin Genet.* 2009;76(4):413–416. 10.1111/j.1399-0004.2009.01212.x 19673950

[180] Tram E Ibrahim-Zada I Briollais L: Identification of germline alterations of the mad homology 2 domain of SMAD3 and SMAD4 from the Ontario site of the breast cancer family registry (CFR). *Breast Cancer Res.* 2011;13(4):R77. 10.1186/bcr2926 21835029 PMC3236341

[181] Xu X Li S Xiao X: Sequence variations of GRM6 in patients with high myopia. *Mol Vis.* 2009;15:2094–2100. 19862333 PMC2765235

[182] Pink AE Simpson MA Desai N: Mutations in the γ-secretase genes NCSTN, PSENEN, and PSEN1 underlie rare forms of hidradenitis suppurativa (acne inversa). *J Invest Dermatol.* 2012;132(10):2459–2461. 10.1038/jid.2012.162 22622421

[183] Chen LJ Tam PO Tham CC: Evaluation of SPARC as a candidate gene of juvenile-onset primary open-angle glaucoma by mutation and copy number analyses. *Mol Vis.* 2010;16:2016–2025. 21042566 PMC2965575

[184] Chen L Qin S Xie J: Genetic polymorphism analysis of CYP2C19 in Chinese Han populations from different geographic areas of mainland China. *Pharmacogenomics.* 2008;9(6):691–702. 10.2217/14622416.9.6.691 18518848

[185] Liu J Zhou X Shan Z: The association of LRP5 gene polymorphisms with ankylosing spondylitis in a Chinese Han population. *J Rheumatol.* 2011;38(12):2616–2618. 10.3899/jrheum.111117 21885484

[186] Deen PM Dahl N Caplan MJ: The aquaporin-2 water channel in autosomal dominant primary nocturnal enuresis. *J Urol.* 2002;167(3):1447–1450. 10.1016/S0022-5347(05)65341-4 11832768

[187] Bonafé L Giunta C Gassner M: A cluster of autosomal recessive spondylocostal dysostosis caused by three newly identified DLL3 mutations segregating in a small village. *Clin Genet.* 2003;64(1):28–35. 10.1034/j.1399-0004.2003.00085.x 12791036

[188] Megremis S Mitsioni A Mitsioni AG: Nucleotide variations in the *NPHS2* gene in Greek children with steroid-resistant nephrotic syndrome. *Genet Test Mol Biomark.* 2009;13(2):249–256 10.1089/gtmb.2008.0083 19371226

[189] Rogan P Mucaki E: Population Fitness and Genetic Load of Single Nucleotide Polymorphisms Affecting mRNA splicing. *ArXiv11070716 Q-Bio* 2011 Reference Source

[190] Day IN Kralovicova J Gaunt TR: IDDM2 locus: 5’ noncoding intron I splicing and translational efficiency effects of INS -23HphI - more than a tag for the INS promoter VNTR. HUGO's 11th Human Genome Meeting (HGM2006), Helsinki Finland.20062011 Reference Source

[191] Taube JR Sperle K Banser L: PMD patient mutations reveal a long-distance intronic interaction that regulates PLP1/DM20 alternative splicing. *Hum Mol Genet.* 2014;23(20):5464–5478. 10.1093/hmg/ddu271 24890387 PMC4168831

[192] Luquin N Yu B Trent RJ: An analysis of the entire SOD1 gene in sporadic ALS. *Neuromuscul Disord.* 2008;18(7):545–552. 10.1016/j.nmd.2008.04.013 18504130

[193] Ueffing N Singh KK Christians A: A single nucleotide polymorphism determines protein isoform production of the human c-FLIP protein. *Blood.* 2009;114(3):572–579. 10.1182/blood-2009-02-204230 19439735

[194] Batty JA Hall AS White HL: An investigation of CYP2D6 genotype and response to metoprolol CR/XL during dose titration in patients with heart failure: a MERIT-HF substudy. *Clin Pharmacol Ther.* 2014;95(3):321–330. 10.1038/clpt.2013.193 24193112

[195] Chiu CF Tsai MH Tseng HC: A novel single nucleotide polymorphism in XRCC4 gene is associated with oral cancer susceptibility in Taiwanese patients. *Oral Oncol.* 2008;44(9):898–902. 10.1016/j.oraloncology.2007.11.007 18164646

[196] Zhao P Zou P Zhao L: Genetic polymorphisms of DNA double-strand break repair pathway genes and glioma susceptibility. *BMC Cancer.* 2013;13:234. 10.1186/1471-2407-13-234 23663450 PMC3655843

[197] Drögemüller C Philipp U Haase B: A noncoding melanophilin gene (MLPH) SNP at the splice donor of exon 1 represents a candidate causal mutation for coat color dilution in dogs. *J Hered.* 2007;98(5):468–473. 10.1093/jhered/esm021 17519392

[198] Kölsch H Jessen F Wiltfang J: Influence of SORL1 gene variants: association with CSF amyloid-beta products in probable Alzheimer’s disease. *Neurosci Lett.* 2008;440(1):68–71. 10.1016/j.neulet.2008.05.049 18541377

[199] Cox DG Crusius JB Peeters PH: Haplotype of prostaglandin synthase 2/cyclooxygenase 2 is involved in the susceptibility to inflammatory bowel disease. *World J Gastroenterol.* 2005;11(38):6003–6008. 16273614 10.3748/wjg.v11.i38.6003 PMC4436724

[200] Thompson D Easton DF: Breast Cancer Linkage Consortium. Cancer Incidence in BRCA1 mutation carriers. *J Natl Cancer Inst.* 2002;94(18):1358–1365. 10.1093/jnci/94.18.1358 12237281

[201] Palomino-Doza J Rahman TJ Avery PJ: Ambulatory blood pressure is associated with polymorphic variation in P2X receptor genes. *Hypertension.* 2008;52(5):980–985. 10.1161/HYPERTENSIONAHA.108.113282 18852390

[202] Xiong Y Wang M Fang K: A systematic genetic polymorphism analysis of the CYP2C9 gene in four different geographical Han populations in mainland China. *Genomics.* 2011;97(5):277–281. 10.1016/j.ygeno.2010.11.004 21126569

[203] Mao M Skogh E Scordo MG: Interindividual variation in olanzapine concentration influenced by UGT1A4 L48V polymorphism in serum and upstream FMO polymorphisms in cerebrospinal fluid. *J Clin Psychopharmacol.* 2012;32(2):287–289. 10.1097/JCP.0b013e31824997a8 22388157

[204] Hiller M Huse K Szafranski K: Phylogenetically widespread alternative splicing at unusual GYNGYN donors. *Genome Biol.* 2006;7(7):R65. 10.1186/gb-2006-7-7-r65 16869967 PMC1779574

[205] Pasvolsky R Feigelson SW Kilic SS: A LAD-III syndrome is associated with defective expression of the Rap-1 activator CalDAG-GEFI in lymphocytes, neutrophils, and platelets. *J Exp Med.* 2007;204(7):1571–1582. 10.1084/jem.20070058 17576779 PMC2118641

[206] Cartault F Nava C Malbrunot AC: A new *XPC* gene splicing mutation has lead to the highest worldwide prevalence of xeroderma pigmentosum in black Mahori patients. *DNA Repair (Amst).* 2011;10(6):577–585. 10.1016/j.dnarep.2011.03.005 21482201

[207] Wang J Sönnerborg A Rane A: Identification of a novel specific CYP2B6 allele in Africans causing impaired metabolism of the HIV drug efavirenz. *Pharmacogenet Genomics.* 2006;16(3):191–198. 16495778 10.1097/01.fpc.0000189797.03845.90

[208] Gaedigk A Bhathena A Ndjountché L: Identification and characterization of novel sequence variations in the cytochrome P4502D6 ( *CYP2D6*) gene in African Americans. *Pharmacogenomics J.* 2005;5(3):173–182. 10.1038/sj.tpj.6500305 15768052 PMC1440720

[209] Green RC Berg JS Grody WW: ACMG recommendations for reporting of incidental findings in clinical exome and genome sequencing. *Genet Med.* 2013;15(7):565–574. 10.1038/gim.2013.73 23788249 PMC3727274

[210] Garcia-Gonzalez MA Jones JG Allen SK: Evaluating the clinical utility of a molecular genetic test for polycystic kidney disease. *Mol Genet Metab.* 2007;92(1–2):160–167. 10.1016/j.ymgme.2007.05.004 17574468 PMC2085355

[211] Leman AR Pearce DA Rothberg PG: Gene symbol: CLN3. Disease: Juvenile neuronal ceroid lipofuscinosis (Batten disease). *Hum Genet.* 2005;116(6):544. 15991331

[212] Keren B Suzuki OT Gérard-Blanluet M: CNS malformations in Knobloch syndrome with splice mutation in *COL18A1* gene. *Am J Med Genet A.* 2007;143A(13):1514–1518. 10.1002/ajmg.a.31784 17546652

[213] Aoyama Y Ozer I Demirkol M: Molecular features of 23 patients with glycogen storage disease type III in Turkey: a novel mutation p.R1147G associated with isolated glucosidase deficiency, along with 9 *AGL* mutations. *J Hum Genet.* 2009;54(11):681–686. 10.1038/jhg.2009.100 19834502

[214] Kwong AKY Fung CW Chan SY: Identification of *SCN1A* and *PCDH19* mutations in Chinese children with Dravet syndrome. *PLoS One.* 2012;7(7):e41802. 10.1371/journal.pone.0041802 22848613 PMC3405017

[215] Li L Xiao X Li S: Detection of variants in 15 genes in 87 unrelated Chinese patients with Leber congenital amaurosis. *PLoS One.* 2011;6(5):e19458. 10.1371/journal.pone.0019458 21602930 PMC3094346

[216] Caridi G Dagnino M Dalgic B: Analbuminemia Zonguldak: case report and mutational analysis. *Clin Biochem.* 2008;41(4–5):288–291. 10.1016/j.clinbiochem.2007.11.016 18154732

[217] Papp J Kovacs ME Olah E: Germline *MLH1* and *MSH2* mutational spectrum including frequent large genomic aberrations in Hungarian hereditary non-polyposis colorectal cancer families: implications for genetic testing. *World J Gastroenterol.* 2007;13(19):2727–2732. 10.3748/wjg.v13.i19.2727 17569143 PMC4147123

[218] Saeed S Bonnefond A Manzoor J: Novel *LEPR* mutations in obese Pakistani children identified by PCR-based enrichment and next generation sequencing. *Obesity (Silver Spring).* 2014;22(4):1112–1117. 10.1002/oby.20667 24319006

[219] Soran H Charlton-Menys V Hegele R: Proteinuria and severe mixed dyslipidemia associated with a novel *APOAV* gene mutation. *J Clin Lipidol.* 2010;4(4):310–313. 10.1016/j.jacl.2010.06.004 21122665

[220] Sznajer Y Coldéa C Meire F: A *de novo* *SOX10* mutation causing severe type 4 Waardenburg syndrome without Hirschsprung disease. *Am J Med Genet A.* 2008;146A(8):1038–1041. 10.1002/ajmg.a.32247 18348267

[221] Eichers ER Green JS Stockton DW: Newfoundland rod-cone dystrophy, an early-onset retinal dystrophy, is caused by splice-junction mutations in *RLBP1*. *Am J Hum Genet.* 2002;70(4):955–964. 10.1086/339688 11868161 PMC379124

[222] Dua-Awereh MB Shimomura Y Kraemer L: Mutations in the desmoglein 1 gene in five Pakistani families with striate palmoplantar keratoderma. *J Dermatol Sci.* 2009;53(3):192–197. 10.1016/j.jdermsci.2008.11.005 19157795 PMC3986861

[223] Hampson G Konrad MA Scoble J: Familial hypomagnesaemia with hypercalciuria and nephrocalcinosis (FHHNC): compound heterozygous mutation in the claudin 16 ( *CLDN16*) gene. *BMC Nephrol.* 2008;9:12. 10.1186/1471-2369-9-12 18816383 PMC2562370

[224] Yeo G Burge CB: Maximum entropy modeling of short sequence motifs with applications to RNA splicing signals. *J Comput Biol.* 2004;11(2–3):377–394. 10.1089/1066527041410418 15285897

[225] Reese MG Eeckman FH Kulp D: Improved splice site detection in Genie. *J Comput Biol.* 1997;4(3):311–323. 10.1089/cmb.1997.4.311 9278062

[226] Beetz C Schüle R Deconinck T: REEP1 mutation spectrum and genotype/phenotype correlation in hereditary spastic paraplegia type 31. *Brain.* 2008;131(Pt 4):1078–1086. 10.1093/brain/awn026 18321925 PMC2841798

[227] Cruchaga C Fernández-Seara MA Seijo-Martínez M: Cortical atrophy and language network reorganization associated with a novel *progranulin* mutation. *Cereb Cortex.* 2009;19(8):1751–1760. 10.1093/cercor/bhn202 19020205

[228] Martoni E Urciuolo A Sabatelli P: Identification and characterization of novel collagen VI non-canonical splicing mutations causing Ullrich congenital muscular dystrophy. *Hum Mutat.* 2009;30(5):E662–672. 10.1002/humu.21022 19309692

[229] Naruse H Ikawa N Yamaguchi K: Determination of splice-site mutations in Lynch syndrome (hereditary non-polyposis colorectal cancer) patients using functional splicing assay. *Fam Cancer.* 2009;8(4):509–517. 10.1007/s10689-009-9280-6 19685281

[230] Pelucchi S Mariani R Trombini P: Expression of hepcidin and other iron-related genes in type 3 hemochromatosis due to a novel mutation in transferrin receptor-2. *Haematologica.* 2009;94(2):276–279. 10.3324/haematol.13576 19144662 PMC2635389

[231] Bacci C Sestini R Provenzano A: Schwannomatosis associated with multiple meningiomas due to a familial *SMARCB1* mutation. *Neurogenetics.* 2010;11(1):73–80. 10.1007/s10048-009-0204-2 19582488

[232] Torregrossa R Anglani F Fabris A: Identification of GDNF gene sequence variations in patients with medullary sponge kidney disease. *Clin J Am Soc Nephrol.* 2010;5(7):1205–1210. 10.2215/CJN.07551009 20448065 PMC2893072

[233] Cohen B Chervinsky E Jabaly-Habib H: A novel splice site mutation of *CDHR1* in a consanguineous Israeli Christian Arab family segregating autosomal recessive cone-rod dystrophy. *Mol Vis.* 2012;18:2915–2921. 23233793 PMC3519373

[234] Fasano T Pisciotta L Bocchi L: Lysosomal lipase deficiency: molecular characterization of eleven patients with Wolman or cholesteryl ester storage disease. *Mol Genet Metab.* 2012;105(3):450–456. 10.1016/j.ymgme.2011.12.008 22227072

[235] Pernet C Bessis D Savignac M: Genitoperineal papular acantholytic dyskeratosis is allelic to Hailey-Hailey disease. *Br J Dermatol.* 2012;167(1):210–212. 10.1111/j.1365-2133.2012.10810.x 22229453

[236] Desmet FO Hamroun D Lalande M: Human Splicing Finder: an online bioinformatics tool to predict splicing signals. *Nucleic Acids Res.* 2009;37(9):e67. 10.1093/nar/gkp215 19339519 PMC2685110

[237] Cartegni L Krainer AR: Disruption of an SF2/ASF-dependent exonic splicing enhancer in *SMN2* causes spinal muscular atrophy in the absence of *SMN1*. *Nat Genet.* 2002;30(4):377–384. 10.1038/ng854 11925564

[238] Smith PJ Zhang C Wang J: An increased specificity score matrix for the prediction of SF2/ASF-specific exonic splicing enhancers. *Hum Mol Genet.* 2006;15(16):2490–2508. 10.1093/hmg/ddl171 16825284

[239] Liu HX Zhang M Krainer AR: Identification of functional exonic splicing enhancer motifs recognized by individual SR proteins. *Genes Dev.* 1998;12(13):1998–2012. 10.1101/gad.12.13.1998 9649504 PMC316967

[240] Lou H Li H Yeager M: Promoter variants in the MSMB gene associated with prostate cancer regulate MSMB/NCOA4 fusion transcripts. *Hum Genet.* 2012;131(9):1453–1466. 10.1007/s00439-012-1182-2 22661295 PMC3956317

[241] Cronin CA Gluba W Scrable H: The *lac* operator-repressor system is functional in the mouse. *Genes Dev.* 2001;15(12):1506–1517. 10.1101/gad.892001 11410531 PMC312721

[242] Wang E Dimova N Cambi F: PLP/DM20 ratio is regulated by hnRNPH and F and a novel G-rich enhancer in oligodendrocytes. *Nucleic Acids Res.* 2007;35(12):4164–4178. 10.1093/nar/gkm387 17567613 PMC1919487

[243] Schneider TD Rogan PK: Computational analysis of nucleic acid information defines binding sites.1999 Reference Source

[244] Botta E Nardo T Orioli D: Genotype-phenotype relationships in trichothiodystrophy patients with novel splicing mutations in the *XPD* gene. *Hum Mutat.* 2009;30(3):438–445. 10.1002/humu.20912 19085937

[245] Lietman SA: Preimplantation genetic diagnosis for hereditary endocrine disease. *Endocr Pract.* 2011;17(Suppl 3):28–32. 10.4158/EP11056.RA 21550946

[246] Hellerud C Burlina A Gabelli C: Glycerol metabolism and the determination of triglycerides--clinical, biochemical and molecular findings in six subjects. *Clin Chem Lab Med.* 2003;41(1):46–55. 10.1515/CCLM.2003.009 12636049

[247] Akiyama M Titeux M Sakai K: DNA-based prenatal diagnosis of harlequin ichthyosis and characterization of *ABCA12* mutation consequences. *J Invest Dermatol.* 2007;127(3):568–573. 10.1038/sj.jid.5700617 17082782

[248] Luquin N Yu B Saunderson RB: Genetic variants in the promoter of *TARDBP* in sporadic amyotrophic lateral sclerosis. *Neuromuscul Disord.* 2009;19(10):696–700. 10.1016/j.nmd.2009.07.005 19695877

[249] Koukouritaki SB Poch MT Cabacungan ET: Discovery of novel flavin-containing monooxygenase 3 ( *FMO3*) single nucleotide polymorphisms and functional analysis of upstream haplotype variants. *Mol Pharmacol.* 2005;68(2):383–392. 10.1124/mol.105.012062 15858076

[250] Karaca M Hismi B Ozgul RK: High prevalence of cerebral venous sinus thrombosis (CVST) as presentation of cystathionine beta-synthase deficiency in childhood: molecular and clinical findings of Turkish probands. *Gene.* 2014;534(2):197–203. 10.1016/j.gene.2013.10.060 24211323

[251] Najah M Di Leo E Awatef J: Identification of patients with abetalipoproteinemia and homozygous familial hypobetalipoproteinemia in Tunisia. *Clin Chim Acta.* 2009;401(1–2):51–56. 10.1016/j.cca.2008.11.012 19056372

[252] Funghini S Thusberg J Spada M: Carbamoyl phosphate synthetase 1 deficiency in Italy: clinical and genetic findings in a heterogeneous cohort. *Gene.* 2012;493(2):228–234. 10.1016/j.gene.2011.11.052 22173106

[253] Fei J Chen SY: Splice site mutation-induced alteration of selective regional activity correlates with the role of a gene in cardiomyopathy. *J Clin Exp Cardiol.* 2013; (S12). 10.4172/2155-9880.S12-004

[254] Lee ST Lee J Lee M: Clinical and genetic analysis of Korean patients with congenital insensitivity to pain with anhidrosis. *Muscle Nerve.* 2009;40(5):855–859. 10.1002/mus.21340 19618435

[255] Pagani F Baralle FE: Genomic variants in exons and introns: identifying the splicing spoilers. *Nat Rev Genet.* 2004;5(5):389–396. 10.1038/nrg1327 15168696

[256] Wadt K Choi J Chung JY: A cryptic *BAP1* splice mutation in a family with uveal and cutaneous melanoma, and paraganglioma. *Pigment Cell Melanoma Res.* 2012;25(6):815–818. 10.1111/pcmr.12006 22889334 PMC7453745

[257] Titeux M Mejía JE Mejlumian L: Recessive dystrophic epidermolysis bullosa caused by COL7A1 hemizygosity and a missense mutation with complex effects on splicing. *Hum Mutat.* 2006;27(3):291–292. 10.1002/humu.9406 16470588

[258] Hertecant JL Ben-Rebeh I Marah MA: Clinical and molecular analysis of isovaleric acidemia patients in the United Arab Emirates reveals remarkable phenotypes and four novel mutations in the *IVD* gene. *Eur J Med Genet.* 2012;55(12):671–676. 10.1016/j.ejmg.2012.08.001 22960500

[259] Kang DH Lee DH Hong YH: Identification of a novel splicing mutation in the *ARSA* gene in a patient with late-infantile form of metachromatic leukodystrophy. *Korean J Lab Med.* 2010;30(5):516–520. 10.3343/kjlm.2010.30.5.516 20890085

[260] Bolisetty MT Beemon KL: Splicing of internal large exons is defined by novel *cis*-acting sequence elements. *Nucleic Acids Res.* 2012;40(18):9244–9254. 10.1093/nar/gks652 22790982 PMC3467050

[261] Di Leo E Magnolo L Lancellotti S: Abnormal apolipoprotein B pre-mRNA splicing in patients with familial hypobetalipoproteinaemia. *J Med Genet.* 2007;44(3):219–224. 10.1136/jmg.2006.046359 17158591 PMC2598025

[262] Caminsky N Mucaki E Rogan P: Dataset 1. Dataset for mRNA splicing mutations in genetic disease. *F1000Research.* 2014 Data Source 10.12688/f1000research.5654.1 PMC4329672 25717368

[263] Brown S Shirley B Caminsky N: Splicing Mutation Calculator (splicemc.cytognomix.com): Initial release. *Zenodo.* 2014 Data Source

