# Supplementary material for: Interpretation of mRNA splicing mutations in genetic disease: review of the literature and guidelines for information-theoretical analysis
Source: F1000Res. 2014 Nov 18;3:282. [Version 1] doi: 10.12688/f1000research.5654.1 (PMC4329672; doi:10.12688/f1000research.5654.1)
Supplement: Peer review history

# Peer review: Referee response for version 1

**DOI:** 10.5256/f1000research.6038.r7225

**Published:** 2015-02-09

**Authors:** Klaas Wierenga

**Affiliations:** 1 Department of Pediatrics, University of Oklahoma Health Sciences Center, Oklahoma City, OK, USA

The paper by Caminsky *et al. *is a welcome and timely review of the complexities of pre-mRNA splicing, the relationship between splicing mutations, detection thereof by IT and/or laboratory, and new challenges posed by next generation sequencing.

It is a rather lengthy and somewhat intimidating review, and I can imagine that many readers, even interested ones, may not make it all the way to the end, certainly not in one session. On the other hand, the review paper is likely to reside on the desk of molecular laboratory directors and and other genetics professionals with an interest in the molecular aspects of genetics.

The review is well written, and the order to topics discussed is logical. Maybe the introduction to splicing is a little short, e.g. little space is dedicated to discussing the spliceosome. The review of the various splice 'sensing' software, and the technology underlying these was in depth.

The relationship between IT predicting splice mutations and laboratory studies to confirm the actual results of aberrant splicing was very well done, and the discussion of NMD and other causes of technical issues relating to demonstrating mutant mRNA resulting from splicing mutations was delightful.

The discussion about laboratory standards (also related to ACMG recommendations) regarding splicing was excellent.

Lastly, the discussion of the impact of splicing mutations and IT in the era of large datasets, including NGS was concise and accurate.

In summary, this review ought to be mandatory reading for all genetics professionals in molecular laboratories, incl. those involved in whole exome/genome sequencing.

The figures were well-selected, and the tables were helpful.

One minor remark: I would mention PLP1 as the gene associated with Pelizaeus-Merzbacher disease (p6, R, middle para).

I have read this submission. I believe that I have an appropriate level of expertise to confirm that it is of an acceptable scientific standard.

# Peer review: Referee response for version 1

**DOI:** 10.5256/f1000research.6038.r6917

**Published:** 2014-12-18

**Authors:** Matthias Titeux

**Affiliations:** 1 Paris Descartes - Sorbonne Paris Cité University, Imagine Institute, Paris, France

The manuscript by Caminsky *et al.* reviews the use of Information Theory (IT) based tools to predict splicing and splicing defects and their possible consequences on the matured transcripts. It is well written and well organized to guide the reader.

Following an introduction covering the basics of the splicing mechanism and signals on the pre-mRNA used by the spliceosome to define the exonic and intronic sequences, the authors described the mathematics behind *in silico* prediction and then focus on the use of their tools (ASSEDA, SMC and Shannon pipeline) and their evolution over the past decade. They review the possibilities and limitations of such tools and compare them to other splicing prediction softwares (HSF, SSF, NNsplice, ESEFinder, RESCUE-ESE…).

Over the years, IT-based splicing predictions have made progress and the overall rate of predictions concordant with experimental validations is around 88%. It has thus become a valuable tool for geneticists and molecular biologists. The authors also list the most common mistakes made by researchers while using their tools, and the ways to avoid them. They also stress the difficulties in predicting the consequences of splicing defect in particular cases due to poorly defined ESE/ESS sequences, combinatorial effects of splicing regulatory proteins (SR proteins and hnRNPs) and large exonic sequence which contains a large number of cryptic donor and splice sites and thus their definition is dependent on the binding of these regulatory proteins.

The manuscript is therefore of great importance for people that use such splicing prediction software as it presents their possibilities, limitations and the best way to report the results. Experimentally validated variants, associated with their predictions(should the authors properly report how the prediction was performed) will help to refine the tools.

Such *in silico* tools are even more valuable in a genomic era where large number of variants are identified by deep sequencing (exomes, whole genome sequencing...) some of which being of unknown significance. Adding better splicing defects prediction (apart from the 2 bp most conserved in the natural splice sites) to the filters used in the prioritization pipeline of next generation sequencing projects should be considered.

I therefore recommend the manuscript for indexation without reservations, if small minor issues listed below can be addressed.

Minor issues :

- Figure 5 is not called in the text.
- Since the journal uses a numbered formatting style for the references, please add the number of the reference in sentences like “Smaoui *et al.* 2004 described….” (page 17), since it is easier to find the given reference among over 260.
- In the supplementary table 2, in the column “concordance (Y/N)” there is in some cases a “P” indicated whose meaning is not clear.

I have read this submission. I believe that I have an appropriate level of expertise to confirm that it is of an acceptable scientific standard.
